# An Exploratory Analysis of Firefighter Reproduction through Survey Data and Biomonitoring

**DOI:** 10.3390/ijerph20085472

**Published:** 2023-04-11

**Authors:** Michelle Engelsman, Andrew P. W. Banks, Chang He, Sandra Nilsson, Debbie Blake, Ayomi Jayarthne, Zubaria Ishaq, Leisa-Maree L. Toms, Xianyu Wang

**Affiliations:** 1 Fire and Rescue NSW, Greenacre, NSW 2190, Australia; 2 Queensland Alliance for Environmental Health Sciences (QAEHS), The University of Queensland, Woolloongabba, QLD 4102, Australia; 3 Repromed, Auckland 1050, New Zealand; 4 School of Public Health and Social Work, Faculty of Health, Queensland University of Technology, Kelvin Grove, QLD 4059, Australia

**Keywords:** firefighting, occupational exposure, biomonitoring, semen, breastmilk, blood, urine, reproduction, fertility

## Abstract

Firefighters are occupationally exposed to chemicals that may affect fertility. To investigate this effect, firefighters were recruited to contribute blood, urine, breast milk or semen samples to (1) evaluate chemical concentrations and semen parameters against fertility standards and the general population; (2) assess correlations between chemical concentrations and demographics, fire exposure and reproductive history; and (3) consider how occupational exposures may affect reproduction. A total of 774 firefighters completed the online survey, and 97 firefighters produced 125 urine samples, 113 plasma samples, 46 breast milk samples and 23 semen samples. Blood, urine and breast milk samples were analysed for chemical concentrations (semivolatile organic compounds, volatile organic compounds, metals). Semen samples were analysed for quality (volume, count, motility, morphology). Firefighter semen parameters were below WHO reference values across multiple parameters. Self-reported rates of miscarriage were higher than the general population (22% vs. 12–15%) and in line with prior firefighter studies. Estimated daily intake for infants was above reference values for multiple chemicals in breast milk. More frequent fire incident exposure (more than once per fortnight), longer duration of employment (≥15 years) or not always using a breathing apparatus demonstrated significantly higher concentrations across a range of investigated chemicals. Findings of this study warrant further research surrounding the risk occupational exposure has on reproduction.

## 1. Introduction

Firefighters are occupationally exposed to chemical hazards at fire incidents, within vehicles and fire stations and through the use of contaminated equipment. Even with high levels of personal protective clothing and equipment, chemical exposure still occurs through dermal absorption, inhalation due to off-gassing equipment post fire exposure, inhalation when reduced levels of breathing protection are employed during fire suppression and subsequent exposure through various routes due to cross contamination [1,2,3,4,5]. A recent review has investigated the potential exposures and health effects of a range of chemicals, including some reproductive and developmental effects [6]. The reproductive toxins and endocrine-disrupting chemicals (EDCs) firefighters face occupationally include metals and semivolatile organic compounds (SVOCs) such as polybrominated diphenyl ethers (PBDEs), polycyclic aromatic hydrocarbons (PAHs), polychlorinated biphenyls (PCBs), organophosphate flame esters (OPEs), volatile organic compounds (VOCs), per- and polyfluoroalkyl substances (PFAS), phthalates and organochlorine pesticides (OCPs) [7,8].

In men, such chemicals have been found to impair spermatogenesis, reduce semen quality, induce sperm DNA damage, affect endocrine levels in exposed men and increase the risk of childhood brain and astroglial tumours in their offspring [9,10,11,12]. For females, SVOCs have presented negative associations with fertility, timing of partition, preterm birth, birth weight and size and increased pregnancy loss [13,14]. They have also been found to affect the endocrine markers of ovarian function, increase the risk of premature ovarian dysfunction and lead to early onset menopause, [15,16,17]. SVOCs and metals are known to pass through the placenta and breast milk, though there remains limited data related to developmental effects [18,19,20,21,22].

Data are limited with regards to the potential for additive, synergistic or antagonistic effects of multiple chemical exposures on reproduction. Researchers have subsequently called for additional work to be conducted in this area to better understand the health impacts, particularly with regards to the long-term health of developing foetuses [23,24,25,26,27].

Although research has increasingly focused on firefighter exposure through human biomonitoring (health-related monitoring through body fluids such as blood, urine, breath and hair to determine levels of exposure to environmental pollutants), to our best knowledge, only two previous studies have utilised biomonitoring to assess aspects of firefighter reproduction [28,29]. Studies examining the potential for firefighter reproductive dysfunction due to occupation have predominantly been by means of survey or through assessing occupation and fertility registries for individuals involved, with no epidemiological studies having been undertaken [30,31,32,33,34].

This biomonitoring and reproduction study sits within a greater study considering firefighter exposure. The aims of the current study are to (1) evaluate chemical concentrations and semen parameters against fertility standards and the general population; (2) assess correlations between chemical concentrations and demographics, fire exposure and reproductive history; and (3) consider how occupational exposures may affect reproduction. Much of the literature surrounding firefighter exposure has pertained to male firefighters due to limited access to female firefighters, or due to women representing a small fraction of the cohort studied and therefore being excluded [6,34]. The current study has been shaped around increasing inclusion opportunities for women to ensure a more balanced presentation of male and female firefighters in health studies.

## 2. Materials and Methods

### 2.1. Survey

Ethics approval was granted through the University of Queensland (#2017000255). To engage in the study, firefighters completed an online consent form and subsequent detailed survey capturing information relating to demographics, exposure, employment and reproduction. Firefighters were invited to contribute biological samples (blood and urine, breast milk or semen), and those who did were instructed to complete a further post-contribution study surrounding their most recent fire exposure(s) (i.e., what type of fire incident was attended prior to the sample collection). Participants who elected to provide a biological sample were provided with code names to protect their identity from that point forward. Participants were requested to provide a single sample, although some offered to contribute multiple samples within the study period. Further details are available in the Supplementary Information (SI).

### 2.2. Sample Collection

A group of pathology companies with collection centres in urban, regional and outer regional locations were engaged to collect samples due to the group’s flexibility in coordinating and supporting a geographically broad anonymous study. Ninety-seven firefighters contributed blood (*n* = 113), urine (*n* = 125) and semen (*n* = 23) samples via the pathology centres and breast milk (*n* = 46) samples at home. Firefighters were not required to provide samples in combination, though paired sample contributions were requested (primarily blood and urine, though semen and breast milk contributions were requested to be paired with blood and urine where possible). All blood contributions were provided with a paired urine sample, and 12 urine samples were provided in isolation by 6 firefighters. Nineteen of the twenty men who provided semen samples also provided blood and urine samples, while one provided semen in isolation. Twenty-seven breast milk samples were provided in isolation, with seventeen paired with blood and urine. Four breast milk samples were collected in 2016 as a pilot study analysis, and all other samples were collected between March 2018 and July 2021 (blood, urine, semen and breast milk). Further detailed information surrounding the collection of samples is provided in the Supplementary Information.

### 2.3. Chemical Analysis

This paper reports on the results of 1-hydroxypyrene (1-OH-PYR), metals and VOCs analysed at the SafeWork NSW Chemical Analysis Branch TestSafe Laboratory (TestSafe). Moreover, 1-, 2-hydroxynaphthalene (1-, 2-, OH-NAP); 2-, 3-hydroxyflourene (2-, 3- OH-FLU); 1-, 2-, 4-, 9-hydroxyphenanthrene (1-, 2-, 4-, 9- OH-PHEN); and OPEs, phthalates, PBDEs and PFAS were analysed at the Queensland Alliance for Environmental and Health Sciences (QAEHS) at the University of Queensland. Details surrounding analytical methods utilised (links to methods published elsewhere), limits of detection, matrices used and the list of individual target analytes can be found in Table S1 in the Supplementary Information.

### 2.4. Statistical Analysis

Data were analysed using IBM SPSS Statistics Version 27, Microsoft Excel 2016, GraphPad Prism 9 and Statistics Kingdom 2017. Sample data were checked for completeness, consistency, accuracy and validity. Exclusions were made for selected analyses if datasets were missing or uncertain. Descriptive statistics were performed to summarise the data. Pearson’s correlations (2-tailed) were used to investigate relationships in normally distributed survey data. Correlations with *p*-values lower than 5% (*p* < 0.05) were designated statistically significant. Due to non-normal distributions in biomonitored data, Mann–Whitney U tests were used when comparing biomonitored results from groups between firefighters within the study separated by characteristics such as gender, frequency of exposure, type of fire exposure (structure, vehicle, rubbish, wildfire, etc.), duration of employment and others, as normal distribution was not observed. During statistical analysis, analytes below the limit of detection (LOD) and limit of quantitation (LOQ) were estimated as the LOD or LOQ divided by two. LOQs were provided by TestSafe NSW and LODs by QAEHS. Rather than reduce sensitivity in analysis by utilizing LOQ from QAEHS (as LOD from TestSafe was not available), the use of LOQ or LOD was determined appropriate depending on the laboratory performing the analysis. Only chemicals with a detection frequency of >50% were included in statistical analyses. When comparing this study with average results from other studies reporting on pooled sample results (without the inclusion of creatinine concentrations), the creatinine concentration of 1.304 g·L^−1^ was utilised [35]. This provided only an estimate and did not allow for the variability of creatinine, so caution must be applied when considering results.

## 3. Results and Discussion

### 3.1. Characteristics of Participants

A total of 774 firefighters completed the online survey collecting data surrounding demographic, employment, exposure and reproductive history. A total of 97 contributed biosamples, resulting in 125 urine samples, 113 blood samples, 46 breast milk samples and 23 semen samples. Of those who contributed biosamples, 59 provided reproductive history data, including pregnancy and birth outcomes. Of those who completed the survey only, 382 provided reproductive history data. Reproductive history was only sought from those who selected that they had attempted to have children since becoming firefighters.

There were no statistically significant differences between firefighters in the “contributed a biosample” group vs. the “survey only” group with regards to frequency of exposure or use of self-contained breathing apparatus (SCBA) in any of the following: working structure fires (internal); external fire suppression; overhaul; and vehicle fires. The survey only group presented non-significant lower percentages (range 2–5%) with regards to always wearing SCBAs across fire types than the group who contributed. As such, the 97 participants who contributed biosamples were used to represent the characteristics of firefighters involved in this study.

#### Surveyed Firefighters’ Reproductive History

The characteristics and self-reported reproductive history of those who had or attempted to have children since becoming a firefighter are presented in Table 1.

This study was not specifically designed to compare fertility rates or the overall fecundity of firefighters with the general population [36]. However, we can report that out of all respondents who provided details of their fertility (Table 1), 441 had attempted a pregnancy, of which 86% (*n* = 378) conceived at least one live birth and 9.0% (*n* = 40) were unsuccessful in conceiving. More detailed data would be required to determine how the fertility rates of this occupational cohort compare with the general Australian population. For example, to obtain such data, this survey would have required questions such as time to pregnancy (TTP) and data relating to their partner’s fertility or fertility treatment, which was outside the scope of this study. The Fertility Society of Australia and New Zealand report that approximately 17% of Australian couples are likely to experience infertility, which is defined as unable to achieve a pregnancy within 1 year of unprotected intercourse; however, for many of those, infertility can be treated through intervention [37].

The rates of miscarriage across all pregnancies reported was 24%, taken from survey answers from both female and male firefighters. Rate of miscarriage by gender warrants consideration, as there is a well-established association of pregnancy loss in men with elevated sperm DNA fragmentation as a consequence of several known factors, including increasing male age and exposure to environmental factors [38]. Male firefighters reported a miscarriage rate of 24% and female firefighters reported a miscarriage rate of 22%. These values exceed the estimated rate of miscarriage for women (12–15%) in the general population, with no known comparable value for men [39]. These results are in line with rates of miscarriage noted by female firefighters in the United States [34].

The remaining 333 survey respondents consisted of those of unknown fertility status, having not intentionally planned a conception since employment as a firefighter. It is relevant to note that while the mean age of respondents is 43 y, this dataset may encompass firefighters who have yet to plan a pregnancy and those who have definitively chosen not to.

### 3.2. Exploratory Analysis into Firefighter Semen

Between 2018 and 2021, 20 men contributed 23 semen samples and 21 blood and urine samples within 2 weeks of the associated semen samples (16 of which were provided on the same day). This section is an extension of findings previously published in a brief research letter related to the current study [28].

Semen data were stratified by age (<45 and ≥45 years of age) based around research demonstrating statistically significant reductions in semen and sperm parameters for men in increasing age brackets above 45 years of age [40]. In this study, younger participants (<45 y) presented non-significant lower mean motility (50% vs. 61%), lower rapid progression (40% vs. 53%) and reduced normal morphology (8.7% vs. 12%) when compared with those ≥45 y. Increased frequency of exposure to fire (at least one fire each week versus frequency of fire exposure being greater than one each week) was associated with non-significant mean decreases in morphology (7.8% vs. 12%), volume (2.2 mL vs. 2.8 mL), sperm concentration (80 M/mL vs. 87 M/mL) and total sperm count (150 M/ejaculate vs. 220 M/ejaculate).

Pearson’s correlations demonstrated significant positive correlations (*p* < 0.05) between semen quality and age, rank (firefighter vs. officer) and occupational hygiene (including use of breathing apparatus, frequency of handwashing, showering post-fire and laundering of personal protective equipment). Increased frequency of laundering, the wearing of a breathing apparatus during fire suppression and overhaul and showering post-incident were all found to have positive effects on semen quality (*p* < 0.05). Three firefighters contributed more than one semen sample. These men experienced 10–88% differences in their own semen parameters. Existing literature has reported an elevated risk of male infertility in firefighters compared with reference group [32]. Although the assessment in this study cannot determine any causal relationships between semen quality and occupational factors, our findings warrant further research.

**Figure 1:**
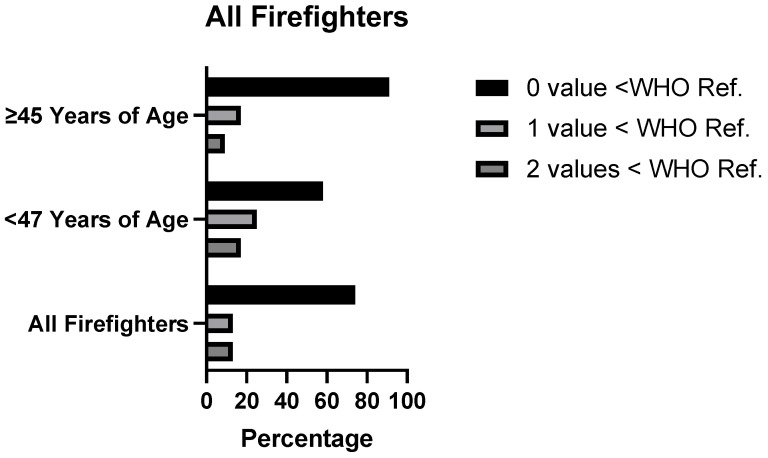
Semen Samples with Combined Parameters Below WHO Reference Values for Fertile Men.

Twenty-six percent of semen samples had sperm concentration, motility and/or morphology value(s) below WHO reference values. This value increased to 42% for those under 45 years of age and decreased to 9% for semen samples from firefighters ≥45 years of age. Findings related to the percentage of firefighters with one or more parameter (sperm concentration, motility and/or normal forms) falling below WHO reference values, with age stratification, are presented in Figure 1.

The prevalence of sperm agglutination, an occurrence wherein motile sperm adhere to each other, was found to be higher in the current study than other published cohorts as shown in Table 2. The rate of sperm agglutination was higher in the younger firefighter cohort.

Seminal volume is known to reduce with age, so it was not unexpected to see that the ≥45 y group was lower than the WHO standards; however, it was unexpectedly low in the younger cohort [43]. In contrast to well-established paradigms regarding semen quality and aging [40,44], this study shows a trend towards higher sperm quality in older participants. This may be associated with older firefighters within this study self-reporting as having an overall lower frequency of fire exposure.

No significant correlations were found between semen parameters and individual chemical concentrations in blood and urine. This may be due to the potential additive and/or interactive effects of the mixture of chemicals firefighters are exposed to, confounding interpretation when considering relationships between seminal parameters and single chemicals [45].

When blood and urine chemical concentrations for firefighters who contributed semen were compared with men who did not contribute semen, very few significant differences (*p* < 0.05) were found across the nearly 100 individual chemicals monitored. The few that were found were in urine and semen include: 1-hydroxy-2-propyl bis (1-chloro-2-propyl) phosphate (BCIPHIPP) (semen median 1.9 µg/g creatinine vs. non-semen 1.2 µg/g creatinine); copper (Cu) (semen median 4.3 µg/g creatinine vs. non-semen 2.0 µg/g creatinine); dimethyl arsinic acid (DMA) (semen median 2.0 µg/g creatinine vs. non-semen 0.72 µg/g creatinine); and arsenobetaine (semen median 48 µg/g creatinine vs. non-semen 71 µg/g creatinine). Some other differences in medians were noted; however, statistical analysis was not run as one or both groups were below the 50% detection frequency. Male firefighters contributing semen were therefore considered statistically similar to male firefighters, and reproductive effects of chemical concentrations is grouped as male and covered in 3.4 Blood and Urine Analysis.

### 3.3. Exploratory Analysis into Firefighter Breast Milk

Forty-six samples were produced from fifteen lactating firefighters. Six women contributed at least two samples, five of which contributed samples after fire incident exposure. An initial analysis was performed on four firefighter breast milk samples contributed in 2016, the other samples were contributed between 2018 and 2020 and analysed in 2022. Between the two analysed sets of samples, there were different limits of detection due to changes in instrumental procedures, and as such, year of analysis will be noted where relevant.

#### 3.3.1. Exploratory Analysis of Chemicals in Breast Milk

When compared with other Australian data reporting on median concentrations in breast milk [46], median firefighter concentrations were higher with regards to the following: 22′44′5-Pentabromodiphenyl ether (BDE-99) (1.1 ng/g lipid, 0.33 ng/g lipid), 22′44′6-Pentabromodiphenyl ether (BDE-100) (0.64 ng/g lipid, 0.57 ng/g lipid) and mirex (0.23 ng/g lipid, 0.12 ng/g lipid) (Table S2 for breast milk results). Median and 95th% levels of tributyl phosphate (TnBP), Tris (2-chloroethyl) phosphate (TCEP), Tris (2-chloroisopropyl) phosphate (TCIPP) and tris (1,3-dichloro-2-propyl) phosphate (TDCIPP) in breast milk far exceeded levels found in 105 women in Beijing [47]. For a full list of chemicals analysed in breast milk, see Table S2.

22′44′-Tetrabromodiphenyl ether (BDE-47) was the dominant congener in both plasma and breast milk. Significant differences were noted with regards to frequency of exposure, with more frequent exposure presenting elevated concentrations of BDE-47, pp-DDE and PCB153 compared with less frequent exposure (see Supplementary Information Section S4: Breast Milk).

Five firefighters provided breast milk samples following two separate fire exposures each, with varying concentrations of chemicals in breast milk suggesting fire exposure may be affecting depuration. For samples provided at 24 h intervals post-fire exposure, a short period of intense fluctuation appeared to follow fire exposure for BDE-47, 22′44′55′-Hexabromodiphenyl ether (BDE-153), TiBP, TCIPP, 2,2′,4,4′,5,5′-hexachlorobiphenyl (PCB153), 2,3,3′,4,4′,5-hexachloro-1,1′-biphenyl (PCB156) and 2,2′,3,4,4′,5,5′-heptachlorobiphenyl (PCB180). This could be denoting a short period of intense depuration, or it could be related to contamination during sample collection, although all procedures possible to prevent such contamination were carried out. It could also be due to uncertainties around the analysis of these compounds from complex matrices such as breast milk. This pattern was not observed for other analysed groups recording levels above the LOD, including TCEP or OCPs. Graphical representation for fluctuations in breast milk for BDE-47, BDE-153, PCBD153 and PCB156 are included in Figures S1–S4.

While some studies have found that not all breastfeeding women demonstrate decreases in chemicals [48,49], other studies demonstrate the stability or general depuration over time for many POPs [50,51,52]. A single prior research study on lactating firefighters in the United States monitoring PBDE concentration and aryl hydrocarbon receptor (AhR) activation found individual variation without a consistent pattern and no significant difference among firefighters following fire exposure [29]. Outside of the current study, no prior studies have monitored lactating women experiencing sporadic, acute exposure over an extended period, including multiple exposures, which may be particularly of note given that the intensity and duration of exposure is likely to play a role in contamination levels.

Although it was outside of our capacity to test collected breast milk for PAHs, given the elevated levels of PAHs in firefighters, it is worth noting potential risks. Urinary 1-hydroxynaphthalene (1-OH-NAP) has been associated with breastmilk, with a 10% increase in 1-naphthol associated with a 1.6% increase in naphthalene in breast milk [53]. Both metabolized and unmetabolized PAHs have been found in the breastmilk of lactating Portuguese women, with phenanthrene and naphthalene (and their metabolites) being amongst the major compounds [22]. PAHs are included in the international list of endocrine-disrupting substances [54], so care should be taken to reduce exposure, where possible.

#### 3.3.2. Exploratory Analysis of Child Health Effects

To understand the contamination of breastmilk in relation to potential child health effects, an assessment of the potential daily intake for an infant (0–6 months) is conducted. This age bracket was selected based around the higher potential for exclusive breast feeding. The calculation of estimated daily intake (EDI) utilized was:

(1) EDI=(CBM×VBM)/BW

where CBM is the concentration for the selected chemical in breast milk, VBM is the average infant daily intake of breast milk, and BW is the average body weight for an infant 0–6 months. For comparison with reference doses (RfD), estimated daily intake (EDI) (ng/kg/day) was calculated using average values of 925 mL of milk per day and an average infant weight of 5.8 kg [55,56].

Several EDI’s were found to be above RfD (see Table 3): BDE-47 (median and 95th%), BDE-99 (median and 95th%) BDE-153 (95th%), TCEP (95th%), TCIPP (median and 95th%), Tris (2-butoxyethyl) phosphate (TBOEP) (median and 95th%) and Tris (2-ethylhexyl) phosphate (TEHP) (median and 95th%). Reference values were unavailable for other chemicals, and even those mentioned may underestimate the risks facing a developing infant [57,58,59]. EDI calculations for chemicals without a known RfD are included in Table S3.

Most toxicological research focuses on exposure to a single agent or analyte; very little research has been undertaken to consider mixed exposures such as those that firefighters and their breast-fed infants may face [58,60]. Furthermore, there exists a lack of information accurately outlining what levels, if any, are specifically safe for infants given their unique susceptibilities.

Infancy is unique in its heightened exposure pathways for lipophilic pollutants, as an infant’s nutritional intake includes a higher lipid ratio than at other stages of life [46]. Although risks of exposure exist and are potentially at their highest in the early weeks of breast feeding due to high infant intake (g/kg body weight), long-term breastfeeding has been proven beneficial to neurodevelopment, with the strong suggestion that the benefits counterbalance the impact of exposure [21,61]. It is important to recognise that if an infant is at risk of exposure through breast milk, it is likely that some exposure has occurred through placental transfer, and therefore, the detoxifying and neurological development aspects of breast milk become more important in ensuring the long-term health of the child [62].

Despite the potential of environmental contaminants in breast milk, it is still the recommended infant feeding method due to its nutritional balance, biologically appropriate composition, promotion of protection against infections, support of immune and neurologic system development and facilitation of maternal–infant attachment [21].

### 3.4. Blood and Urine Analysis

Results of the blood and urine analysis are presented by gender and by matrix in the supplementary information (Tables S4–S7). For statistical analysis, data were grouped (where appropriate) by gender, time since exposure, frequency of exposure, duration of employment, rank (firefighter vs. officer), brigade classification, use of breathing apparatus, biosamples contributed and type of exposure. Correlation between each group and chemical concentration was assessed separately, and thus potential confounding was not considered. Thus, the findings should be interpreted with caution. Statistically significant differences (Mann–Whitney U Test results) are noted in the SI to avoid congestion of reporting within the following results and discussion. The presence of statistically significant differences is noted within each following chemical sub-section. Due to the analysis results suggesting occupational exposure, both median and 95th% concentrations for chemicals biomonitored in blood and urine are reported on, as the exposure that firefighters face when attending incidents varies considerably based around material burnt, duration of exposure, role at the incident and density of smoke [63]. Given the non-normal, right-skewed distribution of chemical concentrations found in firefighter blood and urine, presenting only median without mention of 95th% risks underestimating the risks.

Of the 125 urine samples provided, 24 were outside of WHO confidence ranges with regard to creatinine levels (too dilute) [64]. Even so, given the sensitivity of modern analytical equipment, all samples have been included in the data analysis. Both corrected and uncorrected results have been included in the Supplementary Information (Tables S4–S7).

#### 3.4.1. Polycyclic Aromatic Hydrocarbons (Urine)

Sum hydroxy-naphthalene (ΣOH-NAP) and sum hydroxy-fluorene (ΣOH-FLU) were detected across most groups at frequencies ≥50% and were thus used for statistical comparisons. No statistically significant differences on concentrations of ΣOH-NAP (sum of 1- and 2-hydroxynaphthalene) or ΣOH-FLU (sum of 2- and 3-hydroxyflourene) were noted between types of real fire scenario exposures, possibly due to multiple types of real fires selected by participants for many of the samples, and the ubiquitous nature of PAHs. In real fire scenarios, the median results for ΣOH-NAP (median 5.9 µg/g creatinine, 95th% 19 µg/g creatinine) and ΣOH-FLU (median 0.38 µg/g creatinine, 95th% 1.3 µg/g creatinine) do not appear to exceed concentrations observed in general population studies from Australia (24 µg/g creatinine and 0.51 µg/g creatinine, respectively) [65]. Based on survey responses, less than half of the firefighters who contributed urine for this study did so within 24 h of fire exposure. PAHs can be eliminated from the human system within hours of exposure, which may have limited the potential of finding quantifiable levels [66].

Statistically significant elevations were noted across the urinary PAH results for those exposed to compartment fire behavioural training (CFBT) fires compared with all other fire-exposed groups. CFBT is a method of training to “ensure that firefighters are adequately trained and equipped to perform their roles effectively and safely, to recognise the behaviour of fires, assess conditions in a compartment and make decisions on whether to undertake firefighting in a compartment, and respond appropriately” [67].

Median and 95th% results for firefighters exposed to CFBT in the previous 24 h for ΣOH-NAP (70 µg/g creatinine, 322 µg/g creatinine) and ΣOH-FLU (4.3 µg/g creatinine, 21 µg/g creatinine) exceeded levels of the same who attended real fire scenarios in the previous 24 h (see above). The CFBT group was the only one to present 1-OH-PYR detection frequencies above 50% (median 0.70 µg/g creatinine, 95th% 1.6 µg/g creatinine). The Biological Occupational Exposure Limit (BOEL) for 1-OH-PYR of 1 µg/L (0.77 µg/g creatinine) [68] was exceeded by 50% of CFBT results within 24 h. These results represent high exposure to PAHs that are not necessarily achieved regularly outside of a contrived environment of specific smoke density and duration.

The differences noted between CFBT exposure samples and others were potentially because CFBT exposure was selected in isolation on each occasion (no overlap with other fire exposures) and more likely, as four firefighters provided samples following two closely spaced CFBT fires within a 24 h period prior to collection (see Section S5.1.1 PAHs in Supplementary Information). Findings of fire trainers and firefighters experiencing fire training having higher concentrations of PAHs in urine are not unique to this study [69].

Overall, median male firefighter PAHs in urine were lower than those of the cohort in China, with median 1-hydroxypyrene (1-OH-PYR) (<0.38 µg/g creatinine vs. 0.8 µg/g creatinine), lower firefighter ΣOH-NAP results (3.8 µg/g creatinine vs. 6.2 µg/g creatinine), relatively equivalent ΣOH-FLU results (0.30 µg/g creatinine vs. 4.3 µg/g creatinine) and lower ΣOH-PHE results in firefighters (0.91 µg/g creatinine vs. 5.2 µg/g creatinine) [70]. Firefighters presented with higher sperm concentration (73 million/mL vs. 43 million/mL) and total motility (56% vs. 42%) than the Chinese cohort but were lower for progressive motility (46% vs. 42%), volume (2.0 mL vs. 3.0 mL) and normal forms (9.0% vs. 21%). When CFBT results are considered, firefighters are ~11 × higher for median ΣOH-NAP and equivalent for ΣOH-FLU.

Heavier PAHs have been shown to reduce semen quality, and increased 1-OH-PYR has been positively associated with sperm neck abnormalities, decreased volume and motility [9,71,72]. Prior reproductive studies have found levels of 1-OH-PYR (0.33 ± 0.31 µg/L) to be associated with reduced semen parameters [9], which are lower than what has previously been considered safe (0.5 µg/L) [73]. With an LOQ of 0.5 µg/L, analysis in the current study was limited.

Urinary PAH concentrations approximately equal to those of female firefighters (see Table S6) in the current study have been found to be associated with changes to endocrine markers of ovarian function in women, with other studies supporting similar associations through serum assessment of PAH exposure [74,75,76].

#### 3.4.2. Metals (Whole Blood and Urine)

Higher detection frequency, median and 95th% values for blood lead (Pb) and mercury (Hg) were reported for those not always wearing SCBAs (Pb: 43%, <LOD, 24.9 µg/L and Hg: 50%, 0.75 µg/L, 8.9 µg/L) compared with those always wearing SCBAs (Pb: 9.0%, <LOD, 14 µg/L and Hg:33%, <LOD, 2.5 µg/L), suggesting the importance of occupational hygiene. Statistically significant differences were noted for urinary Cu, selenium (Se), and inorganic arsenic (As) with regard to type of fire exposure, and inorganic As for gender (see Section S5.1.2 Metals in the Supplementary Information).

Firefighters in this study presented with maximum urinary cobalt (Co) levels above what was found to lead to below reference sperm concentrations [77]. The cross-sectional study on Chinese males by Zeng et al. found significant trends for below-reference sperm counts with increasing Se interquartiles (IQs), and it is of note that the Chinese males had much lower Se levels than Australian firefighters (approximately one-third the amount). Increasing Se supported a decrease in abnormal sperm head morphology, and increasing nickel (Ni) was associated with an increasing trend for abnormal sperm head morphology. Firefighter Ni concentrations in urine were approximately half those of Chinese males. Overall, the Chinese males presented with better semen quality than Australian firefighters.

Research has found blood Pb to be related to a moderate alteration in seminal parameters. Although Pb was found present in whole blood in Australian firefighters, its concentration was much lower when compared to results from the literature related to Spanish men [78]. Another Chinese study related to metals in urine showed associations between heavy metals and total sperm motility, progressive motility, or the proportion of normal sperm morphology. Firefighters presented lower median levels of urinary metals to this population for As (6.0 µg/g creatinine vs. 26 µg/g creatinine) and Pb (<LOD vs. 0.68 µg/g creatinine). Firefighter semen (median results) was found to be slightly elevated for motility (56% vs. 49%) and progressive motility (46% vs. 42%), yet had considerably lower normal morphology (9% vs. 21%) [79]. These findings were further supported by Wang et al. (2017) [80].

In a study conducted on 815 pregnant women in Puerto Rico, multiple blood metals were found to act as endocrine disruptors (maternal and foetal), including As, Co, manganese (Mn), nickel (Ni) and Pb [81]. The 95th% results for blood Pb in firefighters in the current study (15 µg/L) were more than double those of the Puerto Rican women (6.4 µg/L), though firefighters’ median levels were lower than Puerto Rican women (<LOD vs. 3.3 µg/L).

#### 3.4.3. Phthalates (Urine)

Within the current study, firefighters with exposure occurring less than 24 h ago presented with significantly lower urinary levels than those with exposure >24 h ago for mono (2-ethylhexyl) phthalate (MEHP) (1.4 vs. 2.0 µg/g creatinine), mono (2-ethyl-5-oxohexyl) phthalate (MEOHP) (1.4 vs. 3.7 µg/g creatinine) and mono (2-ethyl-5-carboxypentyl) phthalate (MECPP) (3.5 vs. 6.8 µg/g creatinine) (*p* < 0.05 for all). Further significant differences were noted within the current study for MEOHP, MEHP and MECPP with regards to type of fire exposure (see Section S5.1.3. Phthalates in the Supplementary Information).

Phthalates have been found to be associated with reduced sperm concentration, straight line velocity, motility, sperm DNA damage, sperm aneuploidy, and increased comet extent even when exposure is below prescribed reference doses [82,83,84]. Firefighter levels reported for IQ3&4 for monoethyl phthalate (MEP) (µg/L) exceeded levels reporting significant reductions in sperm concentration and progressive motility, and firefighter maximum levels for mono(3-carboxypropyl) phthalate (MCPP), which is associated with reduction in sperm motility, exceeded levels in the Chinese population [84].

Median MEP levels in male firefighters (12 µg/g creatinine) exceeded fertile male partners (11 µg/g creatinine) in a Taiwanese study that correlated MEP in urine to that in semen, with a resultant decrease in insulin-like factor 3 [85]. Median female firefighter concentrations for mono-isobutyl phthalate (MiBP) (5.5 µg/g creatinine) exceeded the levels of women found to be experiencing recurrent, unexplained miscarriage (4.2 µg/g creatinine) in a Chinese study [15]. The Ma’anshan Birth Cohort study in China demonstrated that increasing MEP has been associated with a lower concentration of maternal total thyroxine, and when compared with this study, female firefighters presented higher median MEP (11 vs. 7.8 ug/g creatinine) [86].

#### 3.4.4. VOCs (Urine)

Only hippuric acid and mandelic acid (styrene) were detected at a rate of >50%, with 100% of urine samples assessed for styrene returning a positive result. Only the final 10 samples submitted during the study period were analysed for styrene exposure by means of mandelic acid; all prior samples were analysed for ethylbenzene exposure by the same. Statistically significant differences were noted for type of fire exposure as well as gender (see Section S5.1.4. VOCs in the Supplementary Information). Hippuric acid exposure could be due to diets rich in fruits and others [87]; however, three firefighters had levels exceeding 1600 mg/g creatinine [88], all having contributed samples post fire exposure.

Levels in exposed workers at a steel furniture manufacturing company presented with a median level of 800 mg/g creatinine hippuric acid, with unexposed controls presenting 200 mg/g creatinine. Although median concentrations in firefighters (male and female) were in line with unexposed controls, maximum firefighter concentrations were essentially equivalent with those most exposed in the steel furniture manufacturing worker group [89].

Limited data exist around toluene exposure and reproduction, with uncertainty surrounding the possibility of lower-level exposure being associated with miscarriage [90]. Styrene exposure has been found to cause DNA fragmentation in germ cells of Italian male workers facing occupational exposure. The firefighters in this study had considerably lower mandelic acid levels than those in the Italian study, and at this stage, it is unknown whether firefighter concentrations could affect fertility [91].

#### 3.4.5. OPEs (Urine)

When compared to pooled data from the Australian population, concentrations from firefighters are considerably higher in both detection frequency and concentration for bis (2-butoxyethyl) phosphate (BBOEP) (0.87 µg/g creatinine, <LOD of 0.27 µg/g creatinine). The Australian population was higher than firefighters in bis (1,3-dichloroisopropyl) phosphate (BDCIPP) (0.33 µg/g creatinine, 0.17 µg/g creatinine), diphenyl phosphate (DPhP) (34 µg/g creatinine, 0.32 µg/g creatinine) and dibutyl phosphate (DBP) (0.23 µg/g creatinine, 0.08 µg/g creatinine) [92]. OPEs have been previously shown to be an occupational exposure for firefighters in the United States, with female firefighters showing specific OPEs to be up to 5× higher than in the comparison group of female office workers supporting the risks of occupational exposure to OPEs [93].

In the current study, statistically significant differences were measured in urine across groups with regard to bis (methylphenyl) phosphate (BMPP), bis (2-chloroethyl) phosphate (BCEP), BCIPHIPP, DBP, BDCIPP, bis (1-chloroisopropyl) phosphate (BCIPP) and DPhP (see Supplementary Information Section S5.1.5). Other OPEs were not found to present statistically significant differences.

Although research has found OPEs to be associated with reduced male fertility, firefighter urinary levels were below the levels found to cause adverse effects [94,95]. Limited studies suggest OPEs may interfere with endocrine systems, and that exposure has been associated with fertility and pregnancy loss, timing of parturition and preterm birth [96,97]. Overall, reproductive data are lacking for human exposure to OPEs.

#### 3.4.6. PFAS (Plasma)

Within the current study, statistical differences were noted by gender with females presenting significantly higher plasma concentrations of perfluoropentanoic acid (PFPeA) and perfluoropentane sulphonate (PFPeS), while males present significantly higher concentrations of perfluorooctanoic acid (PFOA), perfluorononanoic acid (PFNA), perfluorohexane sulphonic acid (PFHxS and PFHpS) and perfluorooctane sulphonic acid (PFOS). Overall, these findings were reasonable given that females have been found to have reduced concentrations of PFAS in general due to menstruation and lactation [98]. Significantly elevated concentrations were noted for frequency of exposure (PFOS) and longer duration of employment (PFOA, PFOS, PFNA, PFHxS, PFHpS). Those not always wearing SBCAs during smoke diving were statistically elevated for (PFOA, PFNA, perfluorodecanoic acid (PFDA), perfluoroundecanoic acid (PFUnDA), PFHxS, PFHpS and PFOS) than those who reported always wearing SCBAs. Statistical findings are reported in Section S5.1.6. PFAS of the Supplementary Information.

Elevations in mean plasma concentrations became particularly noticeable with the increasing duration of employment, wherein PFHxS increased from 2.7 ± 3.3 µg/L to 5.7 ± 4.8 µg/L for those employed >15 years. The same was observed for total PFOS, where an increase from 4.8 ± 3.4 µg/L to 13.2 ± 14 µg/L was observed in those employed >15 years vs. ≤15 years. Given that aqueous film-forming foams (AFFF) containing PFAS were phased out in the early 2000s from many fire services in Australia, these results are unsurprising [99]. Furthermore, this finding could be influenced by firefighter age, as those employed for >15 years had a mean ± SD age of 53 ± 6.0 years vs. 38 ± 8.4 years for those employed for a shorter duration. A positive association between PFAS concentration and age is also generally observed in the general population [100]. Firefighters were found to have elevated mean plasma concentrations of PFHpA, PFUnDA, perfluorododecanoic acid (PFDoDA), PFPeS, PFHxS, PFHpS and PFOS when compared with the Australian general population, estimated from pooled serum samples collected from the general population in 2016–2017. On the other hand, mean concentrations of PFOA and PFNA were found to be lower than the general population [100]. Firefighter samples were collected between 2018 and 2020 and are therefore not strictly comparable given temporal decreases in the general Australian population, resulting in the magnitude of the elevation potentially being underestimated.

PFAS have been studied with regards to seminal parameters, with mixed findings. Two systematic reviews considering PFAS and male infertility found a lack of consistent results to confirm an association; however, subtle associations between PFOS and lower testosterone or abnormal morphology could not be excluded [101,102].

PFOA has been correlated with longer menstrual cycles, reduced birth size and reduced weight and height [14,103,104]. PFAS have been found to transfer from maternal blood to the placenta [105,106]. PFAS in follicular fluid has been linked to increased risk of some fertility factors [107]. PFHxS has been found to negatively correlate with baseline follicle counts, and upper quartile levels of PFOA and PFOS from NHANES population studies have been found to be associated with anearlier onset of menopause [17]. Studies have found that exposure during developmental windows (pregnancy, pre-puberty) can be key influencers on reproductive outcomes [108]. Firefighters in this study presented with PFAS levels below what has been suggested to affect reproduction.

#### 3.4.7. PBDEs (Plasma)

Within this study, occupational exposure was noted with significant differences between groups with regards to gender, duration of employment and wearing of SCBA. Males presented notably higher detection frequencies and concentrations across all congeners measured in plasma (excluding BDE-99). Females only demonstrated a detection frequency >50% for BDE-47, limiting the ability to undertake statistical comparison between genders.

When comparing duration of employment, only BDE-47 was detected above 50% in those employed for ≤15 years. Detection of BDE-47 in those employed >15 years (median, 95th%: 3.4, 18 ng/g lipid) was statistically significantly greater compared to ≤15 years (median, 95th%: 1.4, 4.0 ng/g lipid). When comparing groups who reported always wearing SCBAs at fire incidents (vehicle, structure fires and overhaul) to those who reported not always wearing BAs, the not always group demonstrated notably higher detection frequencies and concentrations across the interquartile ranges. The group always wearing SCBA was below the 50% detection frequency for all congeners. Further statistical findings are reported in Section S5.1.7 PBDEs of the Supplementary Information.

Conflicting evidence exists surrounding the effects of PBDE exposure on semen quality [11,109,110]. Although studies have demonstrated that elevated levels of BDE-47 in plasma (≥4.4 ng/mL) significantly increase the odds of both indicated and spontaneous preterm birth, female firefighters within this study were below that threshold [111].

### 3.5. Study Strength and Limitations

This study captures a broad-spectrum look at firefighters in real fire situations, thereby providing a snapshot of firefighter exposure outside of prescribed events. However, this presents a wide range of variables surrounding attendance and exposure at real fire scenarios, resulting in levels of uncertainty that cannot be avoided. It does, however, present a more realistic perspective on the average firefighter, even if the current cohort who contributed samples are likely more conscientious than the greater population of firefighters based around survey responses. A strength of this study is the demonstration that firefighters are exposed to many different chemicals. Most studies assessing the health and chemical exposure of firefighters are often just assessing relationships between one compound or group of compounds.

The semen and breast milk segments of this study are presented as exploratory investigations given the limited number of participants and samples. Other lifestyle factors, such as diet, cannot be ruled out as contributing to the study’s findings.

The reproductive element of this study focused only on chemical exposure, with other elements known to cause reproductive distress such as noise, heat, sleep deprivation, physical challenges and psychological traumas being outside of the scope of this study [34]. Furthermore, in this study, only relationships between two variables are assessed (characteristic and chemical concentration) without more detailed assessment of the effect of other variables that may explain the results. For example, when comparing the differences between two groups, ages/gender/diet/health characteristics that may differ in the groups are not directly considered.

This research study did not seek to identify the effects of multiple chemical exposures in firefighters, but rather present that multiple exposures exist within the cohort studied and how such exposures may affect firefighter reproduction.

Limits of quantitation and detection were higher for some chemicals than others, at times above levels found to affect semen parameters in other cohorts or above levels of POPs found in the general population. These factors may contribute to a reduced ability to find statistically significant differences surrounding firefighter exposure across the range of variables imposed and may under-represent the risks. Furthermore, with only LOQ available from TestSafe, the sensitivity of analysis was reduced. The combination of LOQ from TestSafe with the LOD from QAEHS provided a limitation, but was deemed appropriate to support more sensitive analysis, where possible.

## 4. Conclusions

In this study, we show that firefighters experience a broad range of chemical exposures. This research study presents novel data showing that firefighters within this study had reduced quality of semen in comparison to WHO fertility standards, highlighting the need for further research. This study built on prior research to provide a more expanded and novel look at lactating firefighters, investigating a range of chemicals passing through breast milk, calculating estimated daily intake concentrations for breast-fed infants and applying reference doses to provide meaning to those concentrations. This study provides insight into the possible reproductive effects of a range of chemicals biomonitored within firefighter systems and provides important information surrounding the self-reported reproductive history of firefighters. The results highlight the potential for firefighting to negatively affect reproduction for both males and females, as well as the ability for fire-related chemicals to pass through to a breast-fed infant. Our study highlights the broad spectrum of exposure profiles experienced by individual firefighters, which may depend on their occupational and personal hygiene, frequency of exposure, duration of employment and types of fires attended.

## Figures and Tables

**Table 1:** Firefighter Fertility Experiences Reported via Online Survey.

	Contributed a Biosample		Survey Only	
Characteristic	n	%	n	%
Total Participants	97		677	
Male	64		546	
Female	33		131	
Age Mean * ± Standard Deviation	44 ± 11		43 ± 11	
Active Duty (Current Fire Exposure)	91 (94%)		546 (81%)	
Years served Mean * ± Standard Deviation	25 ± 8.5		17 ± 11	
Tobacco Smoker **	3 (3.1%)		48 (7.1%)	
Reported on fertility (% of total surveys in group)	59	61%	382	56%
Naturally conceived at least one child	53	90%	325	85%
Unsuccessful at conceiving	4	7%	36	9%
Unknown cause	11	19%	29	8%
low sperm count	1	2%	20	5%
abnormal sperm	0	0%	7	2%
did not ovulate	1	2%	8	2%
did not menstruate	0	0%	1	0%
hormone imbalance	1	2%	3	1%
other	0	0%	6	2%
Miscarriage ***	14	24%	91	24%
Still Birth	0	0%	5	1%
preterm birth	3	5%	25	7%
gestational diabetes	3	5%	10	3%
low birth weight	3	5%	12	3%
high birth weight	1	2%	4	1%
spina bifida	1	2%	2	1%
congenital heart abnormalities	0	0%	4	1%
club foot	1	2%	2	1%
hydrocephalus, Duane Syndrome, autism spectrum disorder, other neural tube defects	0	0%	2	1%
other physical disabilities	1	2%	6	2%
other	5	8%	31	8%
No, none of these	31	53%	252	66%
Other negative birth outcomes reported included (maximum of one firefighter per group, but could involve multiple children by that individual): cleft pallet, gastroschisis, astigmatisms, attention deficit disorder, hyperactivity disorder, dyspraxia, craniosynostosis, childhood cancer, hyper twisted umbilical cord, dyslexia, encephalocele, cerebral palsy, down syndrome, Trisomy 13, diabetes, oculocutaneous albinism, migraines, tongue tied and jaundice				

* Age and duration of employment data were collected in 5-year increments (employment had one option of <1 year). To calculate the crude mean the midpoint of each bracket was utilised. ** The data of three tobacco smokers excluded in all chemical analysis of biosamples to ensure consistency across analysis and remove potential for confounding factors. *** Miscarriage and multiple miscarriage were two survey options, and some firefighters selected both. To calculate estimated total rate of miscarriage the number of reported miscarriages was added to the number of reported multiple miscarriages, and only one instance was included if both were selected.

**Table 2:** Presence of Sperm Agglutination.

Study	Cohort	*N*	Rate of Agglutination
This Study	Total Firefighters	23	26%
This Study	Age < 45 Years	12	33%
This Study	Age ≥ 45 Years	11	18%
[41]	Infertile men age 20–50	100	18%
[42]	All men via reproductive centre, age not defined	1095	12%

**Table 3:** Estimated Daily Intake (EDI) Values Through Firefighter Breast Milk.

Analyte	RfD	EDI Med (ng/kg/day)	EDI 95th% (ng/kg/day)	Detection Frequency
BDE-47	100	220	630	68%
BDE-99	100	170	220	100%
BDE-153	200	170	630	68%
TCIPP	3600	72,000	420,000	50%
TCEP	2200	*	5200	15%
TBOEP	1500	10,000	14,000	100%

RfD; Reference dose. * These chemicals had detection frequencies below 50% and as such median values were not calculated EDI was calculated by dividing the daily intake of breast milk (925 mL) times the concentration of contaminant in breast milk by body weight (5.8 kg).

## Data Availability

Data related to this study can be obtained from the corresponding author.

## Supplementary Materials

The following supporting information can be downloaded at: https://www.mdpi.com/article/10.3390/ijerph20085472/s1, survey information, sample collection information, statistical significance by chemical group, Table S1: List of Chemicals Analysed by Matrix including LOD/LOQ and Methods, with Abbreviations Included, Table S2 Results of Chemicals Analysed in Breast Milk, Including LODs and Detection Frequencies, Figure S1: Individual Variations in Breast Milk BDE-47 Post Fire Exposure, Figure S2: Individual Variations in Breast Milk BDE-153 Post Fire Exposure, Figure S3: Individual Variations in Breast Milk PCB153 Post Fire Exposure, Figure S4: Individual Variations in Breast Milk PCB156 Post Fire Exposure, Table S3: Calculated Estimated Daily Intake for Breast Fed Infants Based on Chemical Concentrations, Table S4: List of Female Firefighter Blood Results, Including LODs and Detection Frequencies, Table S5: List of Male Firefighter Blood Results, Including LODs and Detection Frequencies, Table S6: List of Female Firefighter Urinary Results, Including LODs and Detection Frequencies, Table S7: List of Male Firefighter Urinary Results, Including LODs and Detection Frequencies.
