# Supplementary material for: An Exploratory Analysis of Firefighter Reproduction through Survey Data and Biomonitoring
Source: Int J Environ Res Public Health. 2023 Apr 11;20(8):5472. doi: 10.3390/ijerph20085472 (PMC10138572; doi:10.3390/ijerph20085472)
Supplement: Supplementary file 1 [file ijerph-20-05472-s001.zip › ijerph-2297548-supplementary.pdf]

An Exploratory Analysis of Firefighter Reproduction through Survey Data and Biomonitoring

Engelsman, Michelle [1,2 *], Andrew P., W. Banks [2], Chang He [2], Sandra Nilsson [2], Debbie Blake [3], Ayomi Jayarthne [2], Zubaria Isha1 [2], Leisa-Maree L. Toms [4], and Xianyu Wang [2]

[1] Fire and Rescue NSW, 1 Amarina Avenue, Greenacre, NSW, 2190
[2] *QAEHS, Queensland Alliance for Environmental Health Sciences, The University of Queensland, 20 Cornwall Street, Woolloongabba, Queensland 4102, Australia*
[3] *Repromed, 105 Remuera Road, Remuera, Auckland, 1050, New Zealand*
[4] *School of Public Health and Social Work, Faculty of Health, Queensland University of Technology, Kelvin Grove, Australia*
* Correspondence: michelle.engelsman@fire.nsw.gov.au

1. Survey:

The recruitment of firefighters commenced by means of information dissemination through multiple gatekeeper organisations. The study was anonymous to support firefighters providing accurate information surrounding personal, reproductive, and occupational hygiene. Stage 1 of the study involved the completion of the survey, which was available to Australian firefighters, aged 18 and over. Stage 2 of the study involved asking participants to contribute human samples. These participants provided an email address by which to be contacted to receive pathology forms, breast milk collection kits, and other study specific information.

Demographic and occupational: 20 questions
Fire exposure, post fire actions, fire station design & hygiene: 27 questions
Reproduction: 8 questions
Breast milk initial survey question: 19 questions

2. Sample Collection:

Firefighters who opted to produce a sample (blood, urine, or semen) for the study were sent deidentified pathology forms. Separate pathology forms were created for blood, urine, and semen contributions outlining what was required from the contribution in each matrix. Firefighters had the option to select from the following: blood & urine, semen, breast milk, or a combination of the three options. Firefighters were told to provide the pathology forms at their choice pathology centre, with all requirements for the phlebotomist outlined on the form. Each firefighter contributing a sample was assigned a unique code name, which was applied to any pathology forms provided to that firefighter. All pathology form were given the same birth date (01/01/1980) to support anonymity, with age range data collected by means of the survey. Breast milk samples were not collected via pathology centre. That breast milk collection method is outlined following the presentation of the blood, urine, and semen collection method.

Firefighters were requested to contribute a total of 40ml of whole blood, 100ml of urine, a minimum of 100ml of breast milk, or an entire semen sample by means of masturbation 3-5 days after last ejaculation, ensuring no condoms, artificial lubricants, or talcs were utilised. Firefighters who contributed blood and urine samples provided 2x20mL of whole blood and 2x50mL of urine as two separate laboratories were engaged in the analysis of the samples. For the blood and urine sent to the Queensland Alliance for Environmental Health Services (QAEHS), 2x10ml lithium heparin tubes of blood were collected, spun and separated into 2x aliquots. They and the urine (50ml container) were then frozen and shipped to the laboratory. For the blood and urine sent to the SafeWork NSW Chemical Analysis Branch, TestSafe Laboraty (TestSafe), 2x10ml lithium heparin whole blood was collected and refrigerated alongside the contributed urine (50ml container) and sent to TestSafe. Blood samples were collected by a trained phlebotomist, urine samples were collected by the firefighter in private.

Firefighters who contributed semen were provided with a sterile, semen specific container for collection by the pathology centre. Firefighters were advised that the sample needed to reach the testing laboratory for analysis within 60 minutes of collection, keeping the sample at body temperature until delivered. Upon receipt at the

pathology centre, samples were maintained at room temperature (20-37°C) until analysis occurred. Analysis occurred in line with Australian National Association of Testing Authorities (NATA) requirements, and via methodology developed based on the World Health Organisation's (WHO) Laboratory Manual for the Examination and Processing of Human Semen, Fifth Edition. Due to the location of participants, 19 samples analysed in New South Wales (NSW) by an automated analyser (SQA-V Gold) and 2 samples were manually analysed in Queensland (QLD).

Analysis of the samples included assessment of viscosity, liquefaction, agglutination, volume, sperm concentration, progressive motile, total motile, immotile, and normal forms. The QLD samples included pH, leukocyte count and immature germ cells.

Firefighters contributing breast milk samples were mailed a collection kit including a sterile jar with markers for volume (a minimum of 100ml requested), a freezer brick, a small esky, and return courier forms for overnight delivery. Firefighters were requested to thoroughly wash hands and any pumps utilised in the collection of expressed milk.

3. Chemicals

Table S1: List of Chemicals Analysed by Matrix including LOD/LOQ and Methods, with Abbreviations Included

Matrix	Chemical(s)	LOD/LOQ	Analytes	Method
Urine	PAHs	0.05µg/L	1-hydroxypyrene (1-OH-PYR)	WCA.158*
		<0.42 µg/L	1-hydroxynaphthalene (1-OH-NAP), 2-hydroxynaphthalene (2-OH-NAP), 2-hydroxyflourene (2-OH-FLU), 3-hydroxyflourene (3-OH-FLU), 1- hydroxyphenanthrene (1-OH-PHEN), 2-hydroxyphenanthrene (2-OH-PHEN), 4- hydroxyphenanthrene (4-OH-PHEN), 9-hydroxyphenanthrene (9-OH-PHEN)	https://doi.org/10.1016/j.envres.2019.109048
	Benzene	0.5 µg/L	s-Phenylmercapturic acid	WCA.211*
	Ethylbenzene & Styrene	0.3 mmol/L	Mandelic Acid	WCA.125*
	Toluene	0.5 mmol/L	Hippuric Acid	WCA.131*
	Xylene	0.5 mmol/L	Toluric Acid	WCA.131*
	Metals	Varying: 0.01 – 40 µmol/L	Antimony (Sb), Beryllium (Be), Bismuth (Bi), Cadmium (Cd), Chromium (Cr), Cobalt (Co), Copper (Cu), Lead (Pb), Manganese (Mn), Mercury (Hg), Nickel (Ni), Selenium (Se), Tellurium (Te), Thallium (Tl), Uranium (U), Vanadium (V)	WCA.215*
	Arsenic	0.02 µmol/L	Monomethyl arsonic acid (MMA), Dimethyl arsinic acid (DMA), Arsenic (III) (As^{III}), Arsenic (V)	WCA.218*

			(As^{V}), Total inorganic arsenic, Arsenobetaine	
	OPEs	<1.360 µg/L	Dibutyl phosphate (DBP), Bis(2-chloroethyl) phosphate (BCEP), Bis(1-chloroisopropyl) phosphate (BCIPP), Bis(methylphenyl) phosphate (BMPP), Bis(2-ethylhexyl) phosphate (BEHP), Diphenyl phosphate (DPhP), Bis(1,3-dichloroisopropyl) phosphate (BDCIPP), Bis(2-butoxyethyl) phosphate (BBOEP), Triphenyl phosphate (TPhP), 2-Ethylhexyl diphenyl phosphate (EHDPP), Tributyl phosphate (TBP), Tris(2-chloroethyl) phosphate (TCEP), Tris(2-chloroisopropyl) phosphate (TCIPP), Tris(methylphenyl) phosphate (TMPP), tris(1,3-dichloroiospropyl) phosphate (TDCPP), 1-Hydroxy-2-propyl bis(1-chloro-2-propyl) phosphate (BCIPHIPP), Tris(2-butoxyethyl) phosphate (TBOEP), Bis(2-butoxyethyl) 3-hydroxyl-2-butoxyethyl phosphate (3OH-TBOEP), Bis(2-butoxyethyl) hydroxyethyl phosphate (BBOEHEP), Tris(2-ethylhexyl) phosphate (TEHP)	https://doi.org/10.1016/j.envint.2017.11.019
	Phthalates	<0.71 µg/L	monomethyl phthalate (MMP), monoethyl phthalate (MEP), mono-isobutyl phthalate (MiBP), mono-butyl phthalate (MnBP), mono(3-carboxypropyl) phthalate (MCPP), monobenzyl phthalate (MBzP), mono(2-ethylhexyl) phthalate (MEHP), mono(2-ethyl-5-oxohexyl) phthalate (MEOHP), mono(2-ethyl-5-hydroxyhexyl) phthalate (MEHHP), mono(2-ethyl-5-carboxypentyl) phthalate (MECPP), monocyclohexyl phthalate (MCHP), mono-n-octyl phthalate (MnOP)	https://doi.org/10.1016/j.envint.2020.105534
Whole Blood	Metals	<0.1µmol/L	Cadmium (Cd), Cobalt (Co), Lead (Pb), Manganese (Mn), Mercury (Hg)	WCA.214*

Plasma	PFAS	<0.0891 µg/L	Perfluorobutanoic acid (PFBA), Perfluoropentanoic acid (PFPeA), Perfluoroheptanoic acid (PFHpA), Perfluorooctanoic acid (PFOA), Perfluorononanoic acid (PFNA), Perfluorodecanoic acid (PFDA), Perfluoroundecanoic acid (PFUnDA), Perfluorododecanoic acid (PFDoDA), Perfluorobutane sulphonic acid (PFBS), perfluoropentane sulphonate (PFPeS), Perfluorohexane sulphonic acid (PFHxS), perfluoroheptanesulfonic acid (PFHpS), Perfluorooctane sulphonic acid (PFOS), linear and branched isomers (Total PFOS), N-Methyl-perfluorooctane sulfonamido acetic acid (NMeFOSAA), perfluorohexanoic acid (PFHxA), perfluorononanesulfonate (PFNS), perfluordecanesulphonate (PFDS), Fluorooctane sulfonamide (FOSA), perfluorooctane sulfonamide acetic acid (FOSAA), N-Ethyl-perfluorooctane sulfonamido acetic acid (NEtFOSAA)	https://doi.org/10.1016/j.ijheh.2019.03.004
Plasma	PBDEs	<0.0078 µg/L	244′-Tribromodiphenyl ether (BDE28), 22′44′-Tetrabromodiphenyl ether (BDE47), 22′44′5-Pentabromodiphenyl ether (BDE99), 22′44′6-Pentabromodiphenyl ether (BDE100), 22′44′55′-Hexabromodiphenyl ether (BDE153)	https://doi.org/10.1016/j.envint.2018.09.014

*Further Workcover details can be found by accessing the Chemical Analysis Branch Handbook via: www.nsw.gov.au/sites/default/files/2022-02/TestSafe-Chemical-Analysis-Branch-Handbook-9th-edition-TS033.pdf

4. Breast Milk

When comparing exposure history within the breast milk group more frequent exposure (≤fortnightly) was significantly higher for the following analytes compared to less frequent exposure (>fortnightly), BDE-47: (U=140, p=0.03677), pp-DDE (U=130, p=0.002857), and PCB153 (U=149, p=0.01363).

Table S2: Results of Chemicals Analysed in Breast Milk, Including LODs and Detection Frequencies

Congener or analyte ng/g lipid	LOD	n	% Detect	Mean ± SD	Min	Max	25th %	50th %	75th %	95th %
BDE-28 (2022)	0.57	30	10%	*	<LOD	4.06	<LOD	<LOD	<LOD	1.41
BDE-28 (2016)	0.21	4	25%	*	<LOD	0.76	<LOD	<LOD	<LOD	0.65
BDE-47 (2022)	0.84	30	63%	1.66 ± 1.46	<LOD	6.33	<LOD	1.22	2.65	4.07
BDE-47 (2016)	0.21	4	100%	3.83 ± 0.91	2.68	4.90	3.51	3.87	4.19	4.76
BDE-99 (2022)	0.05	4	100%	1.13 ± 0.22	0.91	1.43	1.04	1.09	1.17	1.38
BDE-100 (2022)	0.07	4	100%	0.72 ± 0.23	0.56	1.06	0.56	0.64	0.81	1.01
BDE-153 (2022)	0.16	30	63%	1.4 ± 1.49	<LOD	5.59	<LOD	1.12	2.16	4.16
BDE-153 (2016)	0.42	4	100%	1.99 ± 1.12	0.53	3.04	1.41	2.19	2.77	2.99
BDE-154 (2022)	1.07	30	3%	*	<LOD	3.75	<LOD	<LOD	<LOD	<LOD
HCB (2022)	0.36	9	67%	3.29 ± 2.65	<LOD	6.70	<LOD	3.07	5.64	6.30
HCB (2016)	0.10	3	100%	4.31 ± 2.23	2.30	6.70	3.11	3.92	5.31	6.42
pp-DDE (2022)	0.55	26	100%	29.98 ±	6.57	78.04	22.57	26.44	32.41	60.39
pp-DDE (2016)	0.83	4	100%	28.29 ±	10.24	41.54	23.81	30.69	35.17	40.27
pp-DDT (2022)	1.67	26	8%	*	<LOD	5.33	<LOD	<LOD	<LOD	1.99
pp-DDT (2016)	0.21	4	100%	2.88 ± 2.66	1.00	6.80	1.38	1.85	3.35	6.11
mirex (2016)	0.10	4	100%	0.29 ± 0.18	0.14	0.54	0.17	0.23	0.35	0.50
trans-chlordane	0.41	30	13%	*	<LOD	1.93	<LOD	<LOD	<LOD	1.30
β-HCH (2016)	0.21	3	100%	2.18 ± 1.61	0.61	3.83	1.36	2.12	2.97	3.66
PCB28 (2022)	0.25	4	75%	0.5 ± 0.33	<LOD	0.92	0.34	0.48	0.63	0.86
PCB52 (2022)	0.10	4	50%	0.12 ± 0.09	<LOD	0.21	<LOD	0.12	0.19	0.21
PCB101 (2022)	0.56	30	10%	*	<LOD	0.88	<LOD	<LOD	<LOD	0.55
PCB101 (2016)	0.10	4	100%	0.18 ± 0.05	0.12	0.23	0.16	0.18	0.20	0.22
PCB105 (2022)	0.42	30	3%	*	<LOD	<LOD	<LOD	<LOD	<LOD	<LOD
PCB105 (2016)	0.04	4	100%	0.27 ± 0.13	0.12	0.38	0.19	0.30	0.38	0.38
PCB114 (2022)	0.04	4	100%	0.08 ± 0.08	<LOD	0.20	0.04	0.06	0.10	0.18
PCB118 (2022)	1.08	30	17%	*	<LOD	2.1	<LOD	<LOD	<LOD	1.76
PCB118 (2016)	0.10	4	100%	1.46 ± 0.95	0.42	2.7	0.94	1.38	1.9	2.5
PCB138 (2022)	0.90	30	43%	*	<LOD	6.4	<LOD	<LOD	1.3	2.9
PCB138 (2016)	0.06	4	100%	2.74 ± 2.64	0.76	6.6	1.30	1.79	3.2	5.9
PCB153 (2022)	0.65	30	73%	3.2 ± 6.51	<LOD	36	<LOD	1.93	3.0	7.9
PCB152 (2016)	0.03	4	100%	4.05 ± 4.11	1.04	10	1.98	2.51	4.6	9.0
PCB156 (2022)	0.03	30	67%	0.32 ± 0.43	<LOD	1.6	<LOD	0.17	0.34	1.2
PCB156 (2016)	0.04	4	100%	0.36 ± 0.38	0.07	0.92	0.15	0.22	0.43	0.82
PCB157 (2022)	0.05	30	23%	*	<LOD	0.25	<LOD	<LOD	<LOD	0.16
PCB157 (2016)	0.04	4	75%	0.11 ± 0.09	<LOD	0.23	0.07	0.10	0.14	0.21
PCB167 (2022)	0.32	30	3%	*	<LOD	0.20	<LOD	<LOD	<LOD	<LOD
PCB167 (2016)	0.05	4	50%	0.08 ± 0.08	<LOD	0.19	<LOD	0.05	0.11	0.18
PCB180 (2022)	1.61	30	37%	*	<LOD	8.2	<LOD	<LOD	1.7	3.1
PCB180 (2016)	0.10	4	100%	2.27 ± 2.5	0.59	6.0	1.1	1.2	2.4	5.3
PCB189 (2022)	0.16	34	18%	*	<LOD	0.20	<LOD	<LOD	<LOD	<LOD
TPhP (2016)	6.20	4	100%	12.58 ± 6.5	7.70	22	8	10.30	15	21
TBP (2016)	0.94	4	100%	26.5 ± 8.7	16.00	35	21	27.50	33	35
TiBP (2022)	1207	16	25%	*	<LOD	2794	<LOD	<LOD	826	2021
TnBP (2022)	71.91	16	6%	*	<LOD	101.86	<LOD	<LOD	<LOD	52.43
TCEP (2022)	15.35	16	19%	*	<LOD	33.55	<LOD	<LOD	<LOD	32.71

TCP (2016)	1.10	4	100%	6.25 ± 1.43	5.00	8.20	5.30	5.90	6.85	7.93
TCIPP (2022)	909	16	63%	964 ± 831	<LOD	2676	<LOD	<LOD	1176	2666
TCIPP (2016)	78.00	4	100%	190 ±	150	220	180	195	205	217
TDCIPP (2016)	1.70	4	75%	5.24 ± 5.35	<LOD	13.00	2.46	3.55	6.33	11.67
TBOEP (2016)	3.90	4	100%	68.25 ±	56.00	88.00	58.25	64.50	74.50	85.30
TEHP (2016)	21.00	4	75%	57.38 ±	<LOD	86.00	51.38	66.50	72.50	83.30
PFOA	0.01	4	100%	0.06 ± 0.01	0.05	0.07	0.06	0.07	0.07	0.07
PFNA	0.02	4	50%	0.02 ± 0.01	<LOD	0.03	<LOD	0.02	0.03	0.03
PFUnDA	0.02	4	100%	0.1 ± 0.04	0.06	0.14	0.08	0.09	0.10	0.14
PFDoDA	0.03	4	25%	*	<LOD	0.05	<LOD	<LOD	<LOD	0.04
PFTriDA	0.02	4	25%	*	<LOD	0.28	<LOD	<LOD	0.08	0.24
PFTeDA	0.08	4	25%	*	<LOD	0.17	<LOD	<LOD	0.07	0.15
PFHxS	0.00	4	75%	0.03 ± 0.03	<LOD	0.06	0.01	0.03	0.04	0.06
PFOS (linear)	0.02	4	100%	0.2 ± 0.05	0.13	0.26	0.19	0.21	0.23	0.26
PFOS (branched, semi-quantification)	0.02	4	100%	0.14 ± 0.11	0.07	0.31	0.08	0.10	0.15	0.28

Figures S1-S4 present graphical representations of the results from these 5 women, providing concentrations of contaminants measured in breast milk relative to time post birth for samples provided within five days following fire exposure. Where more than one sample was provided within the 5-day period, error bars have been included for Mean±SD.

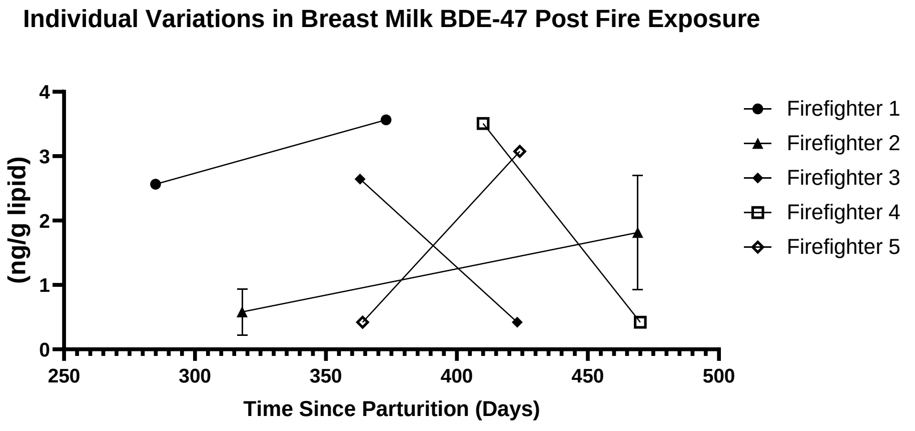

Figure S1: Individual Variations in Breast Milk BDE-47 Post Fire Exposure

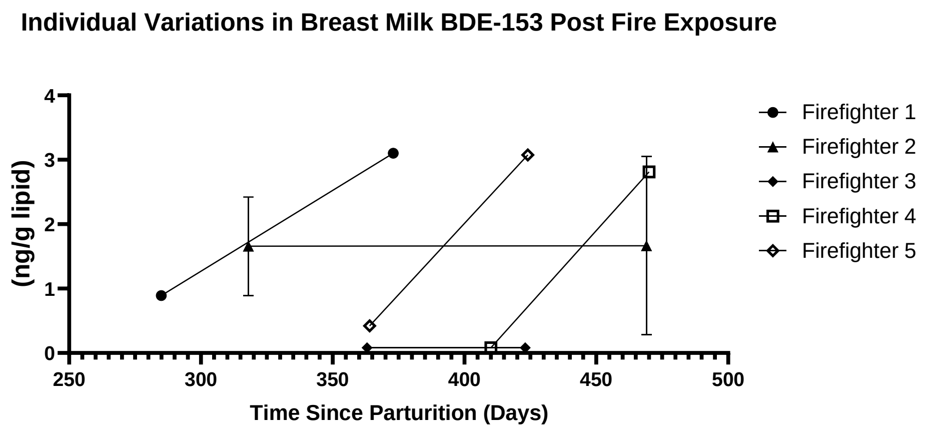

Figure S2: Individual Variations in Breast Milk BDE-153 Post Fire Exposure

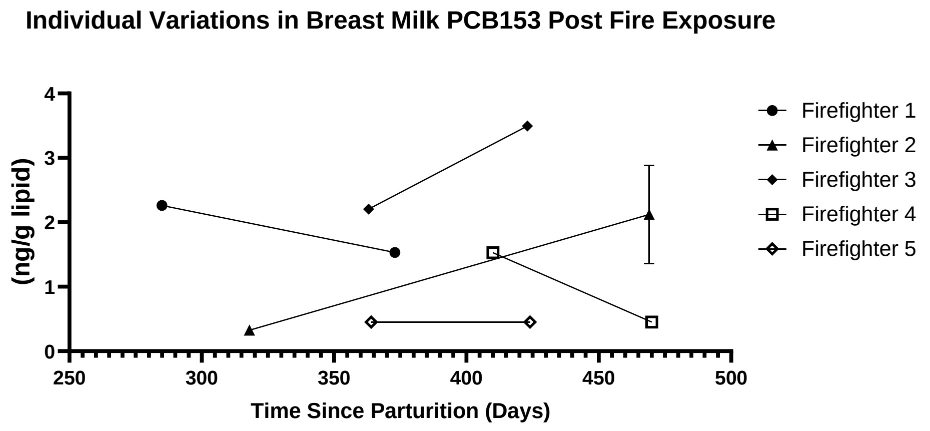

Figure S3: Individual Variations in Breast Milk PCB153 Post Fire Exposure

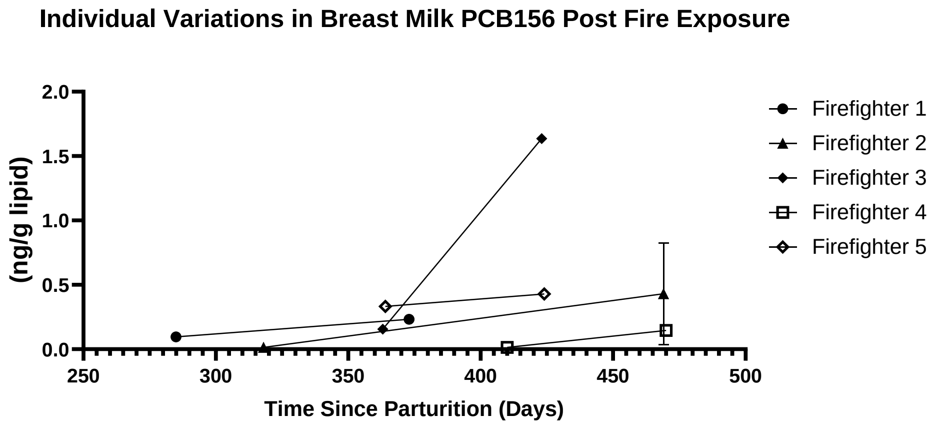

Figure S4: Individual Variations in Breast Milk PCB156 Post Fire Exposure

4.1 Breast Milk EDIs

Table S3: Calculated Estimated Daily Intake for Breast Fed Infants Based on Chemical Concentrations Found in Breast Milk

Analyte	n	RfD*	C_{BM} median ng/mL	C_{BM} 95th% ng/mL	EDI Med	EDI 95th%	Detection Frequency
BDE-28	34		*	1.17	*	186	12%
BDE-47	34	100	1.39	3.96	222	631	68%
BDE-99	4	100	1.09	1.38	173	220	100%
BDE-100	4		0.64	1.01	102	160	100%
BDE-153	34	200	1.08	3.96	171	631	68%
BDE-154	34		*	0.53	*	85	3%
HCB	13		2.82	6.11	450	974	77%
pp-DDE	30		26.56	56.76	4236	9052	100%
pp-DDT	30		*	4.00	*	638	20%
trans-chlordane	34		*	1.59	*	254	15%
PCB28	4		0.48	0.86	76	137	75%
PCB52	4		0.12	0.21	19	33	50%
PCB101	34		*	0.49	*	78	9%
PCB105	34		*	0.24	*	39	12%
PCB114	4		0.06	0.18	9.1	28	100%
PCB118	34		*	1.71	*	273	24%
PCB138	34		0.53	3.85	84	615	50%
PCB153	34		1.93	8.69	308	1386	76%
PCB156	34		0.17	1.22	27	194	71%
PCB157	34		*	0.16	*	26	29%
PCB167	34		*	0.16	*	26	6%
PCB180	34		*	3.37	*	537	41%
PCB189	34		*	0.10	*	16	18%
TCEP	20	2200	*	32.48	*	5181	15%
TCIPP	20	3600	454.42	2662.90	72472	424686	50%
TPhP	4	7000	10.30	20.50	1643	3269	100%
TBP	4		27.50	34.55	4386	5510	100%
TCP	4	1300	5.90	7.93	941	1265	100%
TDCIPP	4		3.55	11.67	566	1860	75%
TBOEP	4	1500	64.50	85.30	10287	13604	100%
TEHP	4	35000	66.50	83.30	10606	13285	75%
PFOA	4		0.07	0.07	10	11	100%
PFNA	4		0.02	0.03	2.8	4.3	50%
PFUnDA	4		0.09	0.14	14	22	100%
PFDoDA	4		*	0.04	*	6.6	25%
PFTriDA	4		*	0.24	*	38	25%
PFTeDA	4		0.04	0.15	6.6	24	25%
PFHxS	4		0.03	0.06	4.5	8.9	75%
PFOS (linear)	4		0.21	0.26	34	41	100%
PFOS (branched, semi-quantification)	4		0.10	0.28	15	44	100%

5. Blood & Urine Analysis

Table S4: List of Female Firefighter Blood Results, Including LODs and Detection Frequencies

Congener or analyte	LOD ug/L	N	% Detect	Unit	Min	25th %	50th %	75th %	Max
∑PBDE	0.0497*	32	6%	ng/g lipid	<LOD	<LOD	<LOD	<LOD	33.00
BDE28	0.001	32	16%	ng/g lipid	<LOD	<LOD	<LOD	<LOD	0.92
BDE47	0.00076	32	56%	ng/g lipid	<LOD	<LOD	0.86	2.23	16.69
BDE99	0.0027	32	13%	ng/g lipid	<LOD	<LOD	<LOD	<LOD	8.67
BDE100	0.0016	32	16%	ng/g lipid	<LOD	<LOD	<LOD	<LOD	3.42
BDE153	0.0078	32	6%	ng/g lipid	<LOD	<LOD	<LOD	<LOD	3.91
PFBA	0.0162	33	79%	ng/mL	<LOD	0.03	0.06	0.10	0.42
PFPeA	0.0414	33	79%	ng/mL	<LOD	0.18	0.45	0.61	1.09
PFHpA	0.0412	33	15%	ng/mL	<LOD	<LOD	<LOD	<LOD	0.10
PFOA	0.0307	33	100%	ng/mL	0.24	0.40	0.88	1.32	5.66
PFNA	0.0318	33	97%	ng/mL	<LOD	0.13	0.26	0.33	1.71
PFDA	0.0387	33	97%	ng/mL	<LOD	0.11	0.15	0.23	0.87
PFUnDA	0.0286	33	97%	ng/mL	<LOD	0.08	0.10	0.14	0.36
PFDoDA	0.0336	33	6%	ng/mL	<LOD	<LOD	<LOD	<LOD	0.14
PFBS	0.0131	33	48%	ng/mL	<LOD	<LOD	<LOD	0.06	0.16
PFPeS	0.0414	33	79%	ng/mL	<LOD	0.18	0.45	0.61	1.09
PFHxS	0.0236	33	100%	ng/mL	0.40	0.64	0.94	1.84	6.08
PFHpS	0.0170	33	67%	ng/mL	<LOD	<LOD	0.06	0.11	0.43
PFOS	0.0891	33	100%	ng/mL	0.69	1.29	2.34	3.42	7.30
Total PFOS*	0.0595	33	100%	ng/mL	1.28	1.70	2.89	4.26	10.23
NMeFOSAA	0.0426	33	12%	ng/mL	<LOD	<LOD	<LOD	<LOD	0.26
Co	0.59	34	12%	μg/L	<LOD	<LOD	<LOD	<LOD	1.00
Pb	20.72	34	3%	μg/L	<LOD	<LOD	<LOD	<LOD	22.79
Mn	5.49	34	94%	μg/L	<LOD	8.38	9.34	11.95	19.78
Hg	1.00	35	34%	μg/L	<LOD	<LOD	<LOD	1.20	6.22
BDE154	0.0250	32	0%						
BDE183	0.0110	32	0%						
PFHxA	0.0236	33	0%						
PFNS	0.0291	33	0%						
PFDS	0.0205	33	0%						
FOSA	0.0166	33	0%						
FOSAA	0.0671	33	0%						
NEtFOSAA	0.0409	33	0%						
8:2 FTS	0.0437	33	0%						
6:2 FTS	0.0390	33	0%						
4:2 FTS	0.0301	33	0%						
PFECHS	0.2258	33	0%						

Table S5: List of Male Firefighter Blood Results, Including LODs and Detection Frequencies

Congener or analyte	LOD ug/L	N	% Detect	Unit	Min	25th %	50th %	75th %	Max
∑PBDE	0.0497*	59	46%	ng/g lipid	<LOD	<LOD	<LOD	23.28	64.56
BDE28	0.001	59	29%	ng/g lipid	<LOD	<LOD	<LOD	0.31	1.76
BDE47	0.00076	59	78%	ng/g lipid	<LOD	0.73	2.62	4.44	23.22
BDE99	0.0027	59	15%	ng/g lipid	<LOD	<LOD	<LOD	<LOD	3.53
BDE100	0.0016	59	54%	ng/g lipid	<LOD	<LOD	0.72	1.64	7.87
BDE153	0.0078	59	39%	ng/g lipid	<LOD	<LOD	<LOD	12.80	44.39
PFBA	0.0162	62	76%	ng/mL	<LOD	0.03	0.07	0.15	9.00
PFPeA	0.0414	62	55%	ng/mL	<LOD	<LOD	0.21	0.49	1.37
PFHxA	0.0236	62	2%	ng/mL	<LOD	<LOD	<LOD	<LOD	0.18
PFHpA	0.0412	62	8%	ng/mL	<LOD	<LOD	<LOD	<LOD	0.15
PFOA	0.0307	62	100%	ng/mL	0.42	1.22	1.64	2.48	3.86
PFNA	0.0318	62	100%	ng/mL	0.13	0.29	0.39	0.57	2.21
PFDA	0.0387	62	95%	ng/mL	<LOD	0.13	0.19	0.24	0.64
PFUnDA	0.0286	62	89%	ng/mL	<LOD	0.08	0.11	0.19	0.57
PFDoDA	0.0336	62	2%	ng/mL	<LOD	<LOD	<LOD	<LOD	0.05
PFBS	0.0131	62	48%	ng/mL	<LOD	<LOD	<LOD	0.06	0.23
PFPeS	0.0414	62	58%	ng/mL	<LOD	<LOD	0.21	0.49	1.37
PFHxS	0.0236	62	100%	ng/mL	0.34	2.68	3.97	6.14	27.01
PFHpS	0.0170	62	98%	ng/mL	<LOD	0.21	0.35	0.45	1.86
PFOS	0.0891	62	100%	ng/mL	0.84	3.81	5.34	9.44	73.06
Total PFOS*	0.0595	62	100%	ng/mL	0.95	5.14	7.29	12.37	83.32
PFNS	0.0291	62	3%	ng/mL	<LOD	<LOD	<LOD	<LOD	0.39
NMeFOSAA	0.0426	62	10%	ng/mL	<LOD	<LOD	<LOD	<LOD	0.16
Co	0.59	62	8%	µg/L	<LOD	<LOD	<LOD	<LOD	1.06
Pb	20.72	62	42%	µg/L	<LOD	<LOD	<LOD	16.58	80.81
Mn	5.49	62	95%	µg/L	2.75	6.73	8.79	10.99	65.93
Hg	1.00	64	41%	µg/L	0.50	0.50	0.50	2.46	17.85
BDE154ng/glipi	0.0250	32	0%						
BDE183ng/glipi	0.0110	32	0%						
PFDS	0.0205	62	0%						
FOSA	0.0166	62	0%						
FOSAA	0.0671	62	0%						
NEtFOSAA	0.0409	62	0%						
8:2 FTS	0.0437	62	0%						
6:2 FTS	0.0390	62	0%						
4:2 FTS	0.0301	62	0%						
PFECHS	0.2258	62	0%						

Table S6: List of Female Firefighter Urinary Results, Including LODs and Detection Frequencies

Urinary congener or analyte	LOD ug/L	N	% Detect	Unit	Min	25th %	50th %	75th %	Max
1-OH-PYR	0.5	40	10%	µg/L	<LOD	<LOD	<LOD	<LOD	3.19
				µg/g creatinine	<LOD	<LOD	<LOD	<LOD	2.46
ΣNAP	0.51	41	98%	µg/L	<LOD	1.47	4.05	8.05	98.71
				µg/g creatinine	<LOD	3.82	6.63	9.43	56.12
23-OH-FLU	0.16	41	66%	µg/L	<LOD	<LOD	0.26	0.62	9.87
				µg/g creatinine	<LOD	<LOD	0.43	0.89	4.59
ΣPHE	1.06	41	15%	µg/L	<LOD	<LOD	<LOD	<LOD	5.25
				µg/g creatinine	<LOD	<LOD	<LOD	<LOD	5.19
MMP	0.21	23	70%	µg/L	<LOD	<LOD	1.36	2.13	14.37
				µg/g creatinine	<LOD	<LOD	1.65	2.08	10.16
MEP	0.13	23	91%	µg/L	<LOD	5.00	9.75	23.59	194.29
				µg/g creatinine	<LOD	6.03	11.20	27.70	341.96
MiBP	0.71	23	96%	µg/L	<LOD	2.34	4.00	8.43	26.41
				µg/g creatinine	<LOD	3.20	5.52	7.87	30.16
MnBP	0.54	23	96%	µg/L	<LOD	3.62	9.12	14.22	38.02
				µg/g creatinine	<LOD	6.02	8.88	12.87	55.09
MCPP	0.01	23	9%	µg/L	<LOD	<LOD	<LOD	<LOD	6.40
				µg/g creatinine	<LOD	<LOD	<LOD	<LOD	5.77
MbzP	0.46	23	87%	µg/L	<LOD	0.63	1.09	1.53	12.00
				µg/g creatinine	<LOD	0.91	1.18	1.67	5.58
MEHP	0.35	23	100%	µg/L	0.46	0.88	1.64	2.80	13.97
				µg/g creatinine	0.64	1.24	2.10	3.36	12.60
MEOHP	0.11	23	78%	µg/L	<LOD	0.57	2.42	4.51	13.08
				µg/g creatinine	<LOD	0.76	2.77	4.70	11.79
MEHHP	0.19	23	96%	µg/L	<LOD	1.18	3.68	7.53	30.17
				µg/g creatinine	<LOD	1.81	5.17	7.86	27.21
MECPP	0.02	23	100%	µg/L	0.03	1.48	3.61	6.84	16.57
				µg/g creatinine	0.06	1.87	4.54	7.13	18.46
DBP	0.026	33	97%	µg/L	<LOD	0.04	0.05	0.09	0.27
				µg/g creatinine	<LOD	0.06	0.10	0.15	0.42
BCEP	0.041	33	82%	µg/L	<LOD	0.05	0.10	0.19	0.72
				µg/g creatinine	<LOD	0.11	0.17	0.33	0.97
BCIPP	0.474	33	73%	µg/L	<LOD	<LOD	0.83	1.76	186.09
				µg/g creatinine	<LOD	<LOD	1.83	2.48	150.93
BMPP	0.001	33	85%	µg/L	<LOD	0.00	0.01	0.02	0.15
				µg/g creatinine	<LOD	0.01	0.02	0.04	0.16
BEHP	0.129	33	3%	µg/L	<LOD	<LOD	<LOD	<LOD	0.22
				µg/g creatinine	<LOD	<LOD	<LOD	<LOD	0.63
DPhP	0.059	33	61%	µg/L	<LOD	<LOD	0.21	0.42	2.75
				µg/g creatinine	<LOD	<LOD	0.26	0.62	3.04
BDCIPP	0.041	33	52%	µg/L	<LOD	<LOD	0.06	0.38	2.44
				µg/g creatinine	<LOD	<LOD	0.13	0.34	3.50
BBOEP	0.307	33	42%	µg/L	<LOD	<LOD	<LOD	0.92	13.30

				µg/g creatinine	<LOD	<LOD	<LOD	1.13	5.57
TPhP	0.057	33	42%	µg/L	<LOD	<LOD	<LOD	0.10	22.16
				µg/g creatinine	<LOD	<LOD	<LOD	0.41	30.14
EHDPP	0.013	33	52%	µg/L	<LOD	<LOD	0.05	0.13	15.50
				µg/g creatinine	<LOD	<LOD	0.03	0.46	21.07
TBP	1.360	33	21%	µg/L	<LOD	<LOD	<LOD	<LOD	2533.16
				µg/g creatinine	<LOD	<LOD	<LOD	<LOD	3445.17
TCEP	0.059	33	48%	µg/L	<LOD	<LOD	<LOD	2.14	35.50
				µg/g creatinine	<LOD	<LOD	<LOD	2.98	348.67
TCIPP	1.037	33	27%	µg/L	<LOD	<LOD	<LOD	2.02	5231.80
				µg/g creatinine	<LOD	<LOD	<LOD	3.31	7115.38
TDCPP	0.101	33	6%	µg/L	<LOD	<LOD	<LOD	<LOD	307.39
				µg/g creatinine	<LOD	<LOD	<LOD	<LOD	418.06
BCIPHIPP	0.166	33	82%	µg/L	<LOD	0.70	2.05	5.06	194.64
				µg/g creatinine	<LOD	0.84	2.23	7.86	955.91
TBOEP	0.030	33	3%	µg/L	<LOD	<LOD	<LOD	<LOD	7.63
				µg/g creatinine	<LOD	<LOD	<LOD	<LOD	10.37
3OH-TBOEP	0.005	33	73%	µg/L	<LOD	<LOD	0.11	0.27	86.76
				µg/g creatinine	<LOD	<LOD	0.17	0.54	118.00
BBOEHEP	0.001	33	58%	µg/L	<LOD	<LOD	0.02	0.07	94.23
				µg/g creatinine	<LOD	<LOD	0.02	0.27	128.16
TEHP	0.593	33	6%	µg/L	<LOD	<LOD	<LOD	<LOD	8.88
				µg/g creatinine	<LOD	<LOD	<LOD	<LOD	12.08
Cr	1.04	34	9%	µg/L	<LOD	<LOD	<LOD	<LOD	1.45
				µg/g creatinine	<LOD	<LOD	<LOD	<LOD	5.11
Cu	1.27	34	44%	µg/L	<LOD	<LOD	<LOD	5.53	15.89
				µg/g creatinine	<LOD	<LOD	<LOD	7.40	117.03
Ni	1.17	34	15%	µg/L	<LOD	<LOD	<LOD	<LOD	4.67
				µg/g creatinine	<LOD	<LOD	<LOD	<LOD	7.69
Se	31.58	34	35%	µg/L	<LOD	<LOD	<LOD	38.36	209.92
				µg/g creatinine	<LOD	<LOD	<LOD	72.56	201.71
V	2.55	34	15%	µg/L	<LOD	<LOD	<LOD	<LOD	2.04
				µg/g creatinine	<LOD	<LOD	<LOD	<LOD	13.86
DMA	1.50	33	55%	µg/L	<LOD	<LOD	2.25	3.75	11.99
				µg/g creatinine	<LOD	<LOD	3.31	4.45	28.15
Arsenic (V)	1.50	33	3%	µg/L	<LOD	<LOD	<LOD	<LOD	1.87
				µg/g creatinine	<LOD	<LOD	<LOD	<LOD	7.36
Arsenobetaine	1.50	33	58%	µg/L	<LOD	<LOD	2.55	5.32	13.49
				µg/g creatinine	<LOD	<LOD	3.57	7.36	103.03
Mandelic Acid	45.65	36	3%	µg/L	<LOD	<LOD	<LOD	<LOD	224.17
				µg/g creatinine	<LOD	<LOD	<LOD	<LOD	64.63
Hippuric Acid	89.59	36	58%	µg/L	<LOD	<LOD	225.62	310.94	698.78
				µg/g creatinine	<LOD	<LOD	120.98	170.61	497.02
MMA	1.50	33	0%						
Arsenic (III)	1.50	33	0%						
Tellurium	10.21	34	0%						
Thallium	0.82	34	0%						
Cobalt	1.18	34	0%						
Lead	4.14	34	0%						

Mn	1.10	34	0%
Hg	4.01	35	0%
MCHP	0.12	23	0%
MnOP	0.17	23	0%
Toluric Acid	89.59		0%
s-Phenylmercapturic acid			0%

Table S7 List of Male Firefighter Urinary Results, Including LODs and Detection Frequencies

Urinary congener or analyte	LOD ug/L	N	% Detect	Unit	Min	25th %	50th %	75th %	Max
1-OH-PYR	0.5	79	9%	µg/L	<LOD	<LOD	<LOD	<LOD	3.72
				µg/g creatinine	<LOD	<LOD	<LOD	<LOD	2.21
ΣNAP	0.51	77	96%	µg/L	<LOD	1.65	3.00	6.68	837.32
				µg/g creatinine	<LOD	2.27	4.27	11.31	366.44
23-OH-FLU	0.16	77	68%	µg/L	<LOD	<LOD	0.29	0.65	55.30
				µg/g creatinine	<LOD	<LOD	0.31	0.71	21.74
ΣPHE	1.06	77	21%	µg/L	<LOD	<LOD	<LOD	<LOD	37.04
				µg/g creatinine	<LOD	<LOD	<LOD	<LOD	16.21
MMP	0.21	58	29%	µg/L	<LOD	<LOD	<LOD	1.42	17.58
				µg/g creatinine	<LOD	<LOD	<LOD	1.22	12.42
MEP	0.13	58	95%	µg/L	<LOD	5.63	19.36	46.00	871.39
				µg/g creatinine	<LOD	6.83	12.16	35.18	1100.46
MiBP	0.71	58	100%	µg/L	1.20	3.12	6.90	14.85	125.05
				µg/g creatinine	1.19	3.22	5.84	9.09	82.41
MnBP	0.54	58	100%	µg/L	0.90	4.67	10.46	23.35	51.22
				µg/g creatinine	1.38	5.47	8.11	15.11	27.91
MCPP	0.01	58	14%	µg/L	<LOD	<LOD	<LOD	<LOD	14.88
				µg/g creatinine	<LOD	<LOD	<LOD	<LOD	17.67
MbzP	0.46	58	81%	µg/L	<LOD	0.53	1.28	3.97	17.93
				µg/g creatinine	<LOD	0.48	1.12	2.54	10.73
MEHP	0.35	58	100%	µg/L	0.39	1.09	1.82	3.10	8.89
				µg/g creatinine	0.22	1.10	1.56	2.29	4.58
MEOHP	0.11	58	76%	µg/L	<LOD	0.73	3.15	5.82	18.61
				µg/g creatinine	<LOD	0.68	2.63	3.95	8.75
MEHHP	0.19	58	100%	µg/L	0.78	3.17	4.53	8.59	41.02
				µg/g creatinine	0.71	2.63	4.03	6.31	19.09
MECPP	0.02	58	100%	µg/L	0.66	2.81	4.85	8.97	29.00
				µg/g creatinine	0.75	2.63	4.18	6.83	14.81
DBP	0.026	70	93%	µg/L	<LOD	0.04	0.06	0.09	0.47
				µg/g creatinine	<LOD	0.05	0.08	0.11	0.71
BCEP	0.041	70	81%	µg/L	<LOD	0.06	0.11	0.25	0.69
				µg/g creatinine	<LOD	0.09	0.15	0.28	0.76
BCIPP	0.474	70	83%	µg/L	<LOD	0.63	1.51	3.19	71.88
				µg/g creatinine	<LOD	0.92	1.70	3.71	33.27
BMPP	0.001	70	96%	µg/L	<LOD	<LOD	0.03	0.05	0.19

				µg/g creatinine	<LOD	<LOD	0.03	0.05	0.23
BEHP	0.129	70	7%	µg/L	<LOD	<LOD	<LOD	<LOD	1.92
				µg/g creatinine	<LOD	<LOD	<LOD	<LOD	0.89
DPhP	0.059	70	81%	µg/L	<LOD	0.10	0.31	0.84	6.73
				µg/g creatinine	<LOD	0.15	0.35	0.81	2.95
BDCIPP	0.041	70	66%	µg/L	<LOD	<LOD	0.22	0.67	5.02
				µg/g creatinine	<LOD	<LOD	0.18	0.64	4.49
BBOEP	0.307	70	63%	µg/L	<LOD	<LOD	0.94	2.82	10.46
				µg/g creatinine	<LOD	<LOD	0.91	2.05	15.92
TPhP	0.057	70	20%	µg/L	<LOD	<LOD	<LOD	<LOD	0.43
				µg/g creatinine	<LOD	<LOD	<LOD	<LOD	1.35
EHDPP	0.013	70	29%	µg/L	<LOD	<LOD	<LOD	0.05	0.33
				µg/g creatinine	<LOD	<LOD	<LOD	0.04	1.14
TBP	1.360	70	6%	µg/L	<LOD	<LOD	<LOD	<LOD	123.96
				µg/g creatinine	<LOD	<LOD	<LOD	<LOD	405.85
TCEP	0.059	70	34%	µg/L	<LOD	<LOD	<LOD	0.13	61.54
				µg/g creatinine	<LOD	<LOD	<LOD	0.15	320.00
TCIPP	1.037	70	10%	µg/L	<LOD	<LOD	<LOD	<LOD	124.93
				µg/g creatinine	<LOD	<LOD	<LOD	<LOD	204.53
TDCPP	0.101	70	11%	µg/L	<LOD	<LOD	<LOD	<LOD	4.74
				µg/g creatinine	<LOD	<LOD	<LOD	<LOD	14.97
BCIPHIPP	0.166	70	83%	µg/L	<LOD	0.53	1.21	2.31	7.13
				µg/g creatinine	<LOD	0.57	1.37	2.25	7.95
TBOEP	0.030	70	4%	µg/L	<LOD	<LOD	<LOD	<LOD	0.14
				µg/g creatinine	<LOD	<LOD	<LOD	<LOD	0.13
3OH-TBOEP	0.005	70	47%	µg/L	<LOD	<LOD	<LOD	0.22	3.86
				µg/g creatinine	<LOD	<LOD	<LOD	0.21	2.71
BBOEHEP	0.001	70	49%	µg/L	<LOD	<LOD	<LOD	0.03	2.20
				µg/g creatinine	<LOD	<LOD	<LOD	0.06	1.17
TEHP	0.593	70	0%	µg/L	<LOD	<LOD	<LOD	<LOD	<LOD
				µg/g creatinine	<LOD	<LOD	<LOD	<LOD	<LOD
Chromium	1.04	73	8%	µg/L	<LOD	<LOD	<LOD	<LOD	5.28
				µg/g creatinine	<LOD	<LOD	<LOD	<LOD	8.11
Cobalt	1.18	73	3%	µg/L	<LOD	<LOD	<LOD	<LOD	2.77
				µg/g creatinine	<LOD	<LOD	<LOD	<LOD	5.21
Copper	1.27	73	59%	µg/L	<LOD	<LOD	4.64	8.39	22.80
				µg/g creatinine	<LOD	<LOD	3.75	6.74	49.57
Lead	4.14	73	4%	µg/L	<LOD	<LOD	<LOD	<LOD	12.43
				µg/g creatinine	<LOD	<LOD	<LOD	<LOD	18.32
Mn	1.10	73	1%	µg/L	<LOD	<LOD	<LOD	<LOD	1.26
				µg/g creatinine	<LOD	<LOD	<LOD	<LOD	4.86
Ni	1.17	73	27%	µg/L	<LOD	<LOD	<LOD	2.49	65.00
				µg/g creatinine	<LOD	<LOD	<LOD	2.75	36.84
Se	31.58	73	59%	µg/L	<LOD	<LOD	38.10	58.36	157.89
				µg/g creatinine	<LOD	<LOD	37.76	50.95	139.60
Thallium	0.82	73	3%	µg/L	<LOD	<LOD	<LOD	<LOD	1.11
				µg/g creatinine	<LOD	<LOD	<LOD	<LOD	3.61
Vanadium	2.55	73	25%	µg/L	<LOD	<LOD	<LOD	<LOD	2.55
				µg/g creatinine	<LOD	<LOD	<LOD	<LOD	11.26

Hg	4.01	63	5%	μg/L	<LOD	<LOD	<LOD	<LOD	6.13
				μg/g creatinine	<LOD	<LOD	<LOD	<LOD	17.73
MMA	1.50	74	1%	μg/L	<LOD	<LOD	<LOD	<LOD	2.25
				μg/g creatinine	<LOD	<LOD	<LOD	<LOD	6.62
DMA	1.50	74	76%	μg/L	<LOD	1.50	3.03	4.48	14.38
				μg/g creatinine	<LOD	2.14	2.99	4.50	21.53
Arsenic (III)	1.50	74	1%	μg/L	<LOD	<LOD	<LOD	<LOD	4.50
				μg/g creatinine	<LOD	<LOD	<LOD	<LOD	6.62
Arsenic (V)	1.50	74	1%	μg/L	<LOD	<LOD	<LOD	<LOD	8.32
				μg/g creatinine	<LOD	<LOD	<LOD	<LOD	11.31
Arsenobetaine	1.50	74	66%	μg/L	<LOD	<LOD	2.92	9.66	533.14
				μg/g creatinine	<LOD	<LOD	3.93	8.24	462.07
Mandelic Acid (ethylbenzene)	45.65	62	11%	μg/L	<LOD	<LOD	<LOD	<LOD	275.73
				μg/g creatinine	<LOD	<LOD	<LOD	<LOD	162.86
Mandelic Acid (Styrene)	45.65	10	100%	μg/L	37.79	47.65	55.89	83.76	139.88
				μg/g creatinine	65.41	95.78	103.43	162.56	368.68
Hippuric Acid	89.59	73	73%	μg/L	<LOD	<LOD	232.93	403.89	1615.57
				μg/g creatinine	<LOD	<LOD	206.94	493.43	2507.93
Tellurium	10.21	73	0%						
Toluric Acid (xylene)	89.59		0%						
s-Phenylmercapturic acid (Benzene)	2.0		0%						

5.1 Statistical significance by Chemical Group

5.1.1 PAHs (Urine)

Significant differences in ΣOH-NAP (U=3, p<0.01) and ΣOH-FLU (U=3, p<0.01) were found between <24hrs real fire scenarios and <24hrs CFBT wherein the level of exposure due to CFBT fire was statistically higher. Measurable increases were noted in detection frequency and analyte concentration across PAHs in <24hrs CFBT compared with real fire scenarios, in some instances by orders of magnitude with regards to median and max.

5.1.2 Metals

When considering metals in whole blood, only manganese (Mn) appeared above 50% across the groups. As such only Mn is used for statistically significant comparisons, of which none were found. It was of note that lead (Pb) and mercury (Hg) increased in detection frequency in men vs women (42v3% and 41v34%). Pb was found to increase in detection frequency in the more exposed group (39% for ≥fortnightly exposure vs 17% for ≥monthly exposure). The detection frequencies of both Pb and Hg increased in the >15 years service group vs the <15 years service (45v20% and 43%v35% respectively) with descriptive statistics showing longer duration employment led to elevated levels of these metals.

Regarding urinary metal results, those exposed to CFBT fires had statistically significant elevations in uncorrected urine for copper (Cu): <72hrs since exposure vs <72hr since CFBT exposure (U=215, p<0.05); and selenium (Se): <72hrs since structure fire vs <72hr since CFBT exposure (U=67.5, p<0.01). Similiar results were noted when comparing CFBT firefighters with all other firefighters exposed within the prior 24hrs. These differences were no longer apparent when urine was creatinine corrected. CFBT (median 6.4μg/L, maximum 16μg/L) were statistically significant greater for inorganic As compared to structure fires (4.4μg/L, maximum 12μg/L) (U=268, p<0.05); however, the reverse was noted for μg/g creatinine wherein structure fires were higher than CFBT (U=308, p<0.01).

Males had statistically significant greater concentrations of inorganic As in μg/L (U=1528, $p<0.05$); however, the reverse was noted for μg/g creatinine wherein female had higher concentrations than males (U=908.5, $p<0.05$). As(V) was detected in 29% of samples reporting recent vehicle fire exposure, compared with 0-3% for other fire types. Other metals were below 50% across all groups so no statistical comparisons were run.

5.1.3 Phthalates

Within the current study, statistically significant differences were noted across groups with regards to the following metabolites: MEOHP, MEHP and MECPP (both ng/L and creatinine corrected). Firefighters exposed to CFBT within the past 24hrs presented with significantly elevated levels of MEOHP ng/L (median 4.4 vs 0.06ng/L) ($p<0.05$, U=58.5). The same did not hold when creatinine corrected. Firefighters with exposure occurring less than 24hrs ago presented with significantly lower median creatinine corrected (ng/g creatinine) urinary levels of MEHP (1.5 vs 2.0), MEOHP (1.5 vs 5.2), and MECPP (3.7 vs 6.8) than those with exposure >24hrs ago (U=213, $p<0.01$, U=185, $p<0.01$, U=226 , $p<0.05$, respectively). No statistically significant differences were seen between <72hr and >72hrs since exposure, exposure frequency (weekly/fortnightly vs greater than), gender or by specific type of fire.

5.1.4 VOCs

In this study statistically significant differences noted in uncorrected urine samples were not seen in creatinine corrected samples, and visa versa. For example, <24hr CFBT presented higher Hippuric Acid (mg/L) compared to real fire scenario exposure <24hrs (U=310, $p<0.01$), yet the finding did not stand when creatinine corrected. Males were elevated in hippuric acid (mg/L) compared to females (U=670, $p<0.01$), but not when creatinine corrected. Hippuric Acid was greater in <24hrs since exposure than >24hrs since exposure (active duty) for creatinine corrected samples (median 290 vs 200 mg/g creatinine) (U=1760.5, $p<0.05$).

5.1.5 OPFRs

Structure Fires (<72hrs) were significantly elevated compared with CFBT (<72hrs) for BCEP (median 0.10 vs 0.06μg/g creatine respectively) (U=250.5, $p<0.01$) and BCIPHIPP (median 1.6 and 0.81μg/g creatinine respectively) (U=216, $p<0.05$). CFBT (<24hrs) were significantly higher than structure fires (<24hrs) for DBP (median 0.10 vs 0.05ng/mL respectively) (U=85, $p<0.05$), BCIPP (median 3.1 and 2.3ng/mL) (U=76, $p<0.05$), BMPP (median 0.04 vs 0.02ng/mL) (U=75, $p<0.05$) and DPhP (median 1.7 vs 0.29ng/mL) (U=69.5, $p<0.05$).

Concentrations in firefighters who attended rubbish fires were significantly elevated compared with structure fires for DBP (median 0.09 vs 0.05ng/mL) (U=28, $p<0.05$) and structure fires significantly greater than wildland fires for BDCIPP (median 0.34 vs 0.14μg/g cr) (U=196, $p<0.05$). With regards to gender, females were significantly higher than males for BCIPHIPP (2.2 vs 1.4μg/g creatinine and 2.0 vs 1.2ng/mL) (U=1546, $p<0.01$ and U=1454, $p<0.05$), yet males were significantly higher than females for BMPP (0.03 vs 0.01ng/mL) (U=715.5, $p<0.01$). Concentrations for firefighters with increased frequency of exposure (<fortnightly, median 0.06μg/L) were significantly greater than for those with less frequent exposure (≥fortnightly, median 0.05μg/L) for DBP (U=1629, $p<0.05$).

5.1.6 PFAS

Within the current study, statistical differences were noted by gender with females presenting significantly higher plasma concentrations of PFPeA (U=1296, p<005) and PFPeS (U=1289, $p<0.05$), yet males being significantly higher in PFOA (U=428, $p=0.000003371$), PFNA (U=488, $p<0.01$), PFHxS (U=256, $p<0.01$), PFHpS (U=180.5, $p<0.01$), and PFOS (U=322, $p<0.01$).

Statistical differences were noted across frequency of exposure with more frequent exposure (≤fortnightly vs >fortnightly) being significantly higher for PFOS (U=1385, $p<0.05$). Duration of employment also presented significant differences wherein those employed for more than 15 years were significantly elevated for a number of PFAS compared to those with shorter employment timeframes: PFOA (U=675, $p<0.01$), PFNA (U=664, $p<0.01$), PFHxS (U=421, $p<0.01$), PFHpS (U=254.5, $p<0.01$), PFOS (U=337, p0.01).

Statistical differences were noted with regards to wearing SCBA. Those not always wearing a breathing apparatus during smoke diving were found to have statistically significant higher plasma concentrations of several PFAS when compared to those who stated that they were always wearing SCBA: PFOA (U=67, p<0.01), PFNA (U=67, p<0.01), PFDA (U=71, p<0.01), PFUnDA (U=80, p<0.01), PFHxS (U=81, p<0.01), PFHpS (U=77, p<0.01), PFOS (U=93, p<0.01).

5.1.7 PBDEs

For BDE-47, males were statistically significantly higher than females (median, max 2.6, 23 vs median, max 0.86, 17 ng/g lipid respectively) (U-617, p<0.01). When comparing duration of employment, only BDE-47 >15yrs employed was statistically significantly greater compared to ≤15yrs (U=534, p<0.01).
